# FOXM1 Signaling Network Transcriptionally Upregulates Expression of Proteins Involved in Mitotic Progression to Induce High Proliferation and Chromosomal Instability in Androgen Receptor-Low Triple-Negative Breast Cancer

**DOI:** 10.3390/ijms27041823

**Published:** 2026-02-14

**Authors:** Padmashree Rida, Raphael Andreae, Noah Bikhazi, Benecia Jackson, Ivan Wang, Nikita Jinna

**Affiliations:** 1 Science Department, Rowland Hall, Salt Lake City, UT 84102, USA; padmashreerida@rowlandhall.org; 2 John and Marcia Price College of Engineering, University of Utah, Salt Lake City, UT 84112, USA; u1500438@utah.edu; 3 Dornsife College of Letters, Arts, and Sciences, University of Southern California, Los Angeles, CA 90089, USA; nbikhazi@usc.edu; 4 Department of Population Sciences, City of Hope Comprehensive Cancer Center, Duarte, CA 91010, USA; bjackson@coh.org; 5 Emory College of Arts and Sciences, Emory University, Atlanta, GA 30322, USA; ivan.wang3@emory.edu

**Keywords:** kinesins, mitotic, centromeric proteins, ubiquitin-proteasome pathway, aggrephagy, FOXM1, WDR5, ASPM, proliferation, genomic instability, aneuploidy

## Abstract

Triple-negative breast cancer (TNBC), particularly the androgen receptor-low (AR-low) subtype, is one of the most aggressive and hard-to-treat forms of BC, characterized by a high index of proliferation, chromosomal instability (CIN), and high prevalence of TP53 mutations. These features fuel therapy resistance, metastases, and poor clinical outcomes. An integrated framework describing the dysregulated molecular networks that support the pathobiology of AR-low TNBC is lacking. Multiple published studies in breast cancer have previously proposed mechanistic links between TP53 loss, AR-low states, and heightened FOXM1-driven G2/M transcriptional programs, potentially via deregulation of E2F activity, chromatin-associated co-regulators (e.g., ATAD2), and disruption of repressive networks involving p53–p21–DREAM and SPDEF. Additional reports suggest that FOXM1-associated circuitry may be reinforced by chromatin regulators such as WDR5 and by mitotic/spindle factors such as ASPM, including through feedback interactions and condensate-associated transcriptional organization. We previously showed that FOXM1, a master regulator transcription factor, is upregulated and is a biomarker of poor prognosis in AR-low TNBC. In this study, we filtered a set of “TNBC core genes” known to promote transcriptional chaos downstream of FoxM1. We identified a set of 15 cell cycle regulators—including mitotic kinesin motors (KIF14, KIF11, KIF4A, KIF2C, and KIF20A), centromeric proteins (CENPA, CENPO, CENPL, CENPF, and OIP5), and regulators of proteolysis (UBE2C, UBE2S, UBE2T, PSMD14, and TUBA1B). These 15 genes, which were ranked highly among genes overexpressed in TNBC featured prominently in gene signatures of chromosomal instability and were also overexpressed among AR-low TNBCs and TP53-mutant breast tumors. We show that expression of each of these 15 genes correlates positively with proliferation markers (Ki67, PCNA, and MCM2) in TNBC, and that the overexpression of this gene set is associated with shorter relapse-free survival and distinct immune/stromal infiltration patterns. In light of prior work, our findings point to a FOXM1-associated 15-gene signature enriched in AR-low TNBC and associated with the high-proliferation and high-CIN phenotypes of this clinically challenging tumor type. This 15-gene set represents an actionable vulnerability with therapeutic potential for AR-low TNBC and provides a framework for rethinking how to manage highly proliferative, genomically unstable BCs.

## 1. Introduction

Breast cancer (BC) remains the most frequently diagnosed malignancy among women in the United States [1]. Its clinical management relies heavily on molecular classification, which is defined by the presence or absence of estrogen and progesterone receptors (ER and PR, respectively), and Human Epidermal Growth Factor Receptor 2 (HER2) amplification. Four principal BC subtypes are recognized: Luminal A (ER+/PR+/HER2− with Ki67 < 14%), Luminal B (ER+/PR+/−/HER2+ or ER+/PR+/HER2− with Ki67 ≥ 14%), HER2-enriched (ER−/PR−/HER2+), and triple-negative BC (TNBC; ER−/PR−/HER2−) [2]. Targeted therapies have significantly improved outcomes for Luminal and HER2-driven cancers, whereas TNBC lacks such options and continues to be managed largely with chemotherapy, surgery, and radiation [3,4]. Among TNBC patients treated with neoadjuvant chemotherapy, those who fail to achieve a pathological complete response frequently relapse within five years, underscoring the aggressive clinical course of this subtype [5].

The burden of BC is not evenly distributed across populations in the US. Although the overall incidence of BC is comparable between Black and White women in the US, mortality among Black women is 40% higher, a disparity linked in part to the two-fold higher incidence of TNBC in the Black population (38 vs. 19 per 100,000) [6]. TNBC itself is molecularly heterogeneous, and efforts to stratify patients have included gene expression–based classification systems [7,8]. One proposed strategy has been to separate tumors by androgen receptor (AR) expression, given the clinical precedent of AR-targeted therapy in prostate cancer. Yet, depending on the threshold used, 65–88% of TNBCs are AR-negative, placing them into the so-called quadruple-negative BC (QNBC) category [9]. QNBC tumors display distinct biological and clinical features compared to AR-positive TNBC. They are enriched for basal-like phenotypes, harbor elevated rates of TP53 mutations, present at younger ages, and are associated with poor disease-free survival [10,11]. At the molecular level, QNBCs show enhanced chromosomal instability, centrosome amplification, copy number alterations, and dysregulated miRNA networks [12,13]. Moreover, QNBC tumors from African American women exhibit unique gene expression profiles and have been reported to overexpress immune checkpoint molecules such as PD-1, PD-L1, and CTLA-4, relative to tumors from White women [10]. These features both compound the aggressiveness of QNBC and limit therapeutic options, further fueling outcome disparities. Despite its clinical relevance, AR-negative TNBC has received far less mechanistic investigation than other BC subtypes. Accumulating data indicate that AR-deficient tumors harbor unique pathobiology, including distinct regulatory networks, high proliferative indices [14,15], and poor prognosis [16], yet they lack well-validated therapeutic targets [17]. This gap highlights the need to define the molecular mechanisms that sustain their aggressive behavior for therapeutic intervention.

FoxM1 is a master regulatory transcription factor and oncogene that regulates apoptosis, drug resistance, DNA damage repair, stem cell renewal, angiogenesis, metastasis, and mitotic spindle maintenance. Aberrantly high activation of FoxM1-driven signaling is essential for the development and progression of many types of solid tumors, and FoxM1 overexpression is associated with higher tumor stage, aneuploidy, higher growth fraction, radiotherapy and chemotherapy resistance, metabolic reprogramming, angiogenesis, and poorer disease outcomes in diverse cancer types [18,19,20]. FoxM1 is expressed only in proliferating normal cells and in tumor cells [21,22], which makes it a good therapeutic target in cancer types wherein the tumor biology is strongly influenced by FoxM1 upregulation. In BC, FoxM1 upregulation drives various facets of tumorigenesis and disease progression [23]. In AR-low TNBC and breast tumors harboring TP53 mutations, FoxM1 overexpression plays a pivotal role in driving the co-upregulation of centrosome amplification and clustering genes, which results in an aggressive disease course and poor patient outcomes [13]. Loss of TP53 function diminishes expression of p21/CIP1, a key cyclin-dependent kinase (CDK) inhibitor. Under normal conditions, p21 restrains the activities of CDK4/6–Cyclin D and CDK2–Cyclin E complexes, preventing premature phosphorylation of the RB. In TP53-deficient cells, insufficient p21 activity leads to premature hyperphosphorylation and inactivation of RB, and inappropriate activation of G1/S gene transcription. TP53 loss of function also promotes the upregulation of oncogenes such as E2F1 and ATAD2, which drive excessive Cyclin E–CDK2 activity. This CDK2 hyperactivity (i) disrupts the DREAM complex [comprising dimerization partner, RB-like, E2F, and multi-vulval class B (MuvB)] from the promoters of G2/M genes [24,25], and (ii) induces YAP/TEAD-mediated transcription of B-MYB and FOXM1. Concurrently, ATAD2 further promotes B-MYB accumulation and B-Myb–MuvB (MMB) complex formation; the MMB complex then recruits FOXM1, culminating in an abnormally high build-up of FOXM1 at G2/M gene promoters during the S/G2 transition. As cells complete DNA replication, CDK1 activity—relieved from ATR-CHK1 inhibition—is able to phosphorylate FOXM1, with PLK1 providing additional activating phosphorylation events. This dual phosphorylation leads to abnormally high levels of FoxM1 activation at the G2/M boundary. FOXM1 then drives the transcription of genes critical for centrosome amplification and clustering, alongside genes promoting proliferation, drug resistance, and survival, culminating in poorer patient prognosis [13]. Epigenetic mechanisms also contribute to transcriptional upregulation of FoxM1; in TNBC for example, the expression of FOXM1 is upregulated by the WD Repeat Domain 5 (WDR5) protein [26]. WDR5 is a core component of histone methyltransferase complexes that catalyze the trimethylation of histone H3 at lysine 4 (H3K4me3) [27]. Promoters marked by H3K4me3 are typically transcriptionally active, with higher H3K4me3 enrichment correlating with a more open chromatin state [28,29,30]. Additionally, methylation at H3K4 may influence how effector proteins bind, thereby shaping downstream biological processes [31]. In TNBC, additional mechanisms lead to aberrantly high levels of FoxM1: the androgen receptor (AR) normally upregulates SPDEF, a transcription factor that suppresses FOXM1 expression by disrupting FoxM1’s autoregulatory positive feedback loop. This SPDEF-mediated repression is further reinforced by the p53–p21–DREAM axis. However, in AR-deficient or AR-low TNBC—particularly when combined with TP53 mutation—this regulatory network also collapses, causing massive overexpression of FoxM1 and its target genes in AR-low TNBC [13]. Our previous work also uncovered that, because of the above-stated selective advantages conferred by FoxM1 dysregulation, FOXM1 is indispensable for the survival of p53-deficient and AR-low TNBC cells harboring amplified centrosomes [13]. Further elucidation of the nexus of dysregulation around FoxM1 may therefore be critical to understanding the tumor biology of AR-low TNBC and TP53-deficient breast tumors for successful therapeutic intervention.

Recently, weighted gene co-expression network analysis [32] identified three “hub” genes—Abnormal Spindle-Like Microcephaly-Associated gene or ASPM/MCPH5, CDC20, and TTK—which are all known target genes of MMB-FoxM1—whose elevated expression was associated with advanced tumor grades, decreased relapse-free survival (RFS), and lower overall survival (OS). These hub genes strongly correlated with cellular processes such as the cell cycle, DNA replication, homologous recombination, and P53 signaling pathways. Among these “hub” genes, ASPM plays conserved and multifaceted roles in crucial aspects of cell division. In interphase cells, ASPM localizes to centrosomes and the nucleus, and plays an important role in homologous recombination-mediated DNA repair by increasing the half-life of BRCA1; inhibition of ASPM destabilizes BRCA1, impairing the efficiency of DNA double-strand break repair [33] and leading to genomic instability. ASPM also copurifies with the Cyclin E/Cdk2 complex and protects Cyclin E from ubiquitination and proteasome-mediated degradation [34].

Interestingly, a mass spectrometry-based screen for phase-separated, chromatin-associated proteins in breast tumor cells identified FoxM1 as a preeminent candidate [35]. Liquid–liquid phase separation (LLPS) is a fundamental biological process wherein proteins and nucleic acids de-mix from the cellular environment and self-assemble into dense, liquid-like subcellular compartments/condensates or hubs through multi-valent interactions. Xie et al., 2025 [35] showed that in BC cells, the sub-nuclear LLPS of FoxM1 with forkhead box consensus DNA elements, is critical for FoxM1’s oncogenic function, as it allows FoxM1 to achieve a high local concentration and to effectively compartmentalize the transcriptional machinery, preserve chromatin accessibility and super-enhancer landscapes at cycle gene homology region (CHR) promoter sites, thus sustaining high G2/M target gene expression for tumor progression and metastasis. Disrupting this LLPS led to the dissolution of the FoxM1-containing biomolecular condensates, which in turn impaired oncogenic transcription, reduced breast tumor growth, and inhibited metastasis in animal models [35]. Importantly, a genome-wide screen in hepatocellular carcinoma (HCC) cells to identify intranuclear regulators that boost FoxM1’s transactivation of downstream target genes through LLPS [36] found that an isoform of ASPM physically interacts with FOXM1 within the nucleus, leading to the formation of condensates containing both ASPM and FoxM1. This interaction, in turn, enhanced FoxM1 protein’s stability by preventing its ubiquitination and proteasome-mediated degradation. Furthermore, FOXM1 was shown to transcriptionally activate ASPM expression, creating a “double positive feedback loop” where each protein reciprocally promotes the other’s activity and presence. ChIP-sequencing revealed that the great majority of ASPM-annotated genes overlapped with FOXM1-bound genes, indicating that ASPM collaborates with FoxM1 to increase expression of FoxM1 target genes [36]. This coordinated overexpression of ASPM and FoxM1 is strongly linked to poor patient prognosis in HCC [36]. ASPM is upregulated in various cancers (including breast, ovarian, prostate, glioblastoma, and hepatocellular carcinoma). A multicohort study [37] demonstrated a significant association between high ASPM expression and aggressive BC features (such as higher tumor grade, higher mitotic score, increased Ki67 labeling index, poor Nottingham Prognostic Index, and lympho-vascular invasion). In multivariate analyses, high ASPM expression was an independent predictor of poorer BC-specific survival and distant metastasis-free survival, and ASPM overexpression was potentially involved in radiotherapy and chemotherapy resistance. The above-mentioned study thus identified ASPM as a promising prognostic marker and a potential therapeutic target for BC [37]. Across multiple malignancies, elevated ASPM expression is linked to advanced stage, metastasis, and poor prognosis, including in pancreatic [38], liver [39], and brain cancers [40]. Beyond its transcriptional cooperation with FOXM1, ASPM also plays a conserved, FOXM1-independent role in safeguarding genomic integrity. Accurate mitotic spindle orientation depends on coordinated interactions between astral microtubules and the cell cortex, and disruption of this process underlies primary microcephaly (MCPH). ASPM (MCPH5), the most frequently mutated gene in MCPH, recruits citron rho-interacting kinase (CITK) to spindle poles, where their combined activity regulates astral microtubule dynamics required for correct spindle positioning. Disruption of the ASPM–CITK interaction leads to spindle misorientation, premature mitotic exit, and aberrant differentiation of progenitor cells, contributing to defective tissue expansion. Independently, ASPM also forms a complex with the microtubule-severing ATPase katanin, which together controls microtubule minus-end dynamics at spindle poles. ASPM tracks and caps growing microtubule minus ends, recruits katanin to microtubules, and promotes katanin-mediated severing, collectively regulating spindle flux and poleward microtubule turnover. Loss of ASPM–katanin coordination disrupts spindle organization and microtubule dynamics, impairing mitotic fidelity. Thus, ASPM preserves genomic integrity by ensuring proper spindle architecture and chromosome segregation through precise control of microtubule dynamics.

Herein, we investigate the mechanisms that operate downstream of FoxM1 to drive proliferation and genomic instability in AR-low TNBC and TP53-mutant BCs. We focus on three interlinked modules—mitotic kinesin motors, centromeric and kinetochore proteins, and proteolysis regulators—that converge to sustain high proliferation while compromising fidelity of chromosomal segregation. We propose a unifying FoxM1–WDR5–ASPM regulatory axis that orchestrates this dysregulation, and discuss how disruption of this axis fuels tumor evolution, intra-tumoral heterogeneity, and therapy resistance. By framing AR-low TNBC as a disease entity defined by (a) transcriptional chaos, (b) high proliferation, and (c) genomic instability, this study highlights novel actionable vulnerabilities that could guide the development of future therapeutic strategies and improve TNBC outcomes.

## 2. Results

### 2.1. FOXM1-Regulated Cell Cycle Genes That Drive Transcriptional Chaos Promote a Highly Proliferative State in AR-Low TNBC

TNBCs in general exhibit high levels of inter- and intra-tumoral heterogeneity, and this heterogeneity is also manifest in the aspect of proliferation. AR-low TNBC is more proliferative than AR-high TNBCs. AR-high TNBCs have a lower pathologic complete response (pCR) rate (10%) to neoadjuvant chemotherapy, compared to BL1 TNBCs (52%) [5]. To understand more about the patterns of dysregulation underlying the heterogeneity observed in TNBCs, a 2014 study by Radovich et al. compared RNA sequencing transcriptomic data of TNBCs with that of micro-dissected normal breast tissues (from reduction mammoplasty) and histologically normal tumor-adjacent tissues, finding that TNBC heterogeneity is attributable to chaos in transcription [41]. Transcriptional chaos refers to the wide range in the number of dysregulated genes observed when comparing each individual TNBC to the set of normal tissues. Transcriptional chaos generally takes place without compromising the functional status of its drivers—in other words, key drivers of this process are generally not mutated or inactivated, but instead, remain functional and become aberrantly activated. Notably, transcriptional chaos is thought to occur as a downstream consequence of CIN, rather than as a cause, as widespread transcriptional deregulation is often driven by aneuploidy [42]. Studies have shown that transcriptional chaos leads to the generation of a wider range of cell states and proteomic profiles [43]. The consequent emergence of greater populational heterogeneity presents clear selective advantages by increasing tumor cell survival rates in environments where multiple stressors exist. Interestingly, the chaotic dynamics of oscillations and a broadening of the distribution in levels of otherwise tightly and periodically expressed transcription factors (such as the Forkhead box M1 protein, FoxM1) can have differential effects on downstream gene networks, enhance the assembly of functional protein complexes, and may end up, paradoxically, upregulating very specific networks and protein modules [43]. Radovich et al. [41] found that the transcriptional chaos in TNBCs was positively correlated with non-silent DNA mutational load, and chaos analysis identified a network of 146 core cell cycle-regulated genes dysregulated in more than 90% of the TNBCs examined [41]. FoxM1, a “master regulator” transcription factor, was found to directly regulate 61 (42%) of the 146 core genes, and FoxM1 itself was overexpressed 17.2-fold in TNBCs compared to micro-dissected normal tissues [24,41]. Since FoxM1 binds to the promoters of and strongly activates expression of genes critical for G2, proper progression of mitosis, and the M/G1 transition, the authors concluded that FoxM1 overexpression causally drives the profound transcriptional dysregulation that typifies TNBCs.

We reasoned that genes that are associated with and drive heterogeneity in proliferation must be discoverable within this dysregulated network of 146 core genes in TNBCs. To specifically uncover genes whose overexpression drives and mechanistically supports high proliferation in AR-low TNBCs, we filtered the set of 146 “FoxM1-regulated TNBC core genes” for a statistically significant negative correlation between the expression of the core genes and the expression of AR, among TNBCs. We found that the expression of 82 genes of this core set showed a statistically significant negative correlation with the expression of AR among TNBCs (Supplementary Figure S1A–E), meaning that they are overexpressed in AR-low TNBCs. Notably, there was a high degree of positive correlation between the expression of most of these 82 core genes (Supplementary Figure S1A–E), which reflected their co-regulation, directly or indirectly, by FoxM1. We then focused our study on the subset of genes among the 82 (a) that are among the top overexpressed genes in TNBCs, (b) have established roles in the regulation of mitotic progression and cell proliferation, and (c) whose overexpression is associated with poor prognosis. We used the UALCAN platform to determine the overexpression rankings for the 82 genes of interest, among TNBCs in the TCGA dataset. Based on the rationale that overexpression of drivers of proliferation is likely to drive a more aggressive disease course and poorer outcomes, we also performed Cox proportional hazards regression analysis for each gene separately to identify the subset of genes whose overexpression results in a poorer prognosis. Based on these preliminary analyses, we identified a group of 15 genes, including 5 kinesin motor proteins, 5 centromeric proteins, and 5 proteins that play important roles in the proteolysis of cell cycle regulators. We found that these 15 genes were expressed at a statistically significantly higher level among breast tumors that are categorized as “AR-low” compared to “AR-high” breast tumors (Supplementary Figure S2), indicating that the overexpression of these 15 genes is correlated with low AR expression across all breast tumors. We confirmed in two separate databases (METABRIC and Oncohuman) that these 15 genes are also significantly upregulated in AR-low relative to AR-high TNBC tumors (Supplementary Figures S3 and S4). The remainder of this study examined how the overexpression of these 15 cell-cycle regulatory proteins likely supports and provokes high proliferation and chromosomal instability, specifically in AR-low TNBCs, precipitating poor outcomes in this subtype.

### 2.2. A Set of 15 Cell Cycle-Related Proteins That Are Overexpressed in AR-Low TNBC Are Associated with High Proliferation

**Figure 1:**
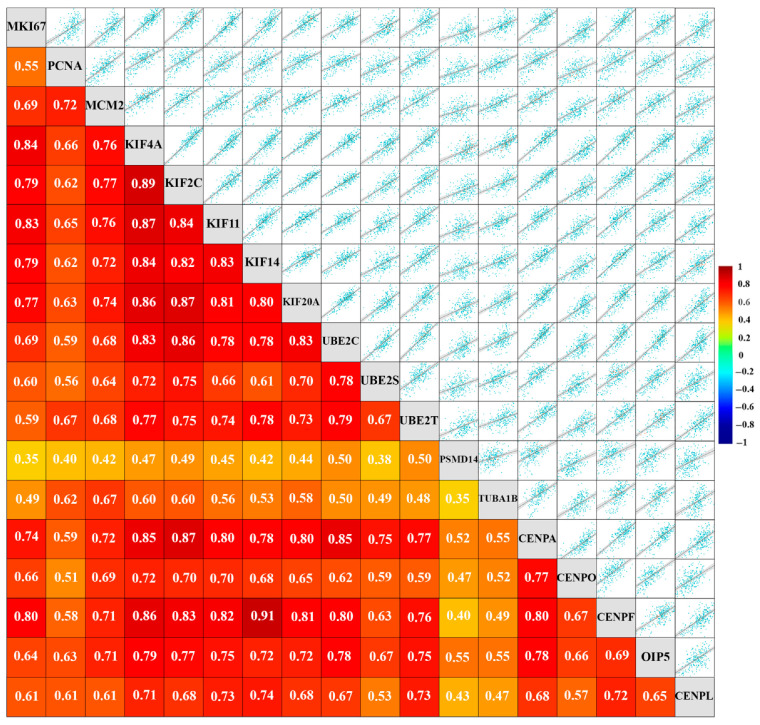
The expression of a set of cell cycle-related proteins that are highly overexpressed in AR-low TNBC is correlated with the expression of established markers of proliferation. “Targeted” gene expression correlation analysis of 15 cell cycle-regulated genes that are overexpressed in AR-low TNBC, and markers of proliferation (MKI67, PCNA, and MCM2; for all RNA sequencing data, TNBC status was determined by immunohistochemistry), performed using the bc-GenExMiner online platform. Scatter plots depict Pearson’s pairwise correlations, and the numbers inside the squares indicate the strength of the observed Pearson’s pairwise correlations. Total *n* = 4421 for each pairwise comparison. Strong negative correlations are depicted in blue, and strong positive correlations are depicted in warm colors. *p*-Values for all pairwise correlations were statistically significant (*p* < 0.0001).

Since deregulated proliferation is a hallmark of cancer [44,45], the measurement of the proliferative fraction of cells in a tumor has prognostic value. To evaluate our hypothesis that the 15 MMB-FoxM1-regulated cell cycle genes identified above are important for driving or supporting the high-proliferation phenotype of AR-low TNBC, we evaluated associations between the expression of the 15 genes of interest and three well-established markers of proliferation in tumors—Ki67, Proliferating Cell Nuclear Antigen (PCNA), and Mini-Chromosome Maintenance 2 (MCM2). The Ki67 antigen is expressed universally in the nuclei of all proliferating cells (normal and tumor) in all phases of the cell cycle, but is not expressed in quiescent cells in the G0 phase [46], making it an excellent marker for the proliferative population within a group of cells [47]. In BC, a strong correlation exists between the percentage of cells positive for Ki67 expression and histologic grade because both parameters are associated with proliferation [48,49]. Furthermore, higher tumor stages and lymph node positivity are associated with a higher percentage of cells expressing Ki67 [49,50,51], which is associated with poor disease-free survival (DFS) and OS [49,52]. Ki67 serves as a dynamic biomarker for choosing the systemic treatment of early-stage BC [53], and changes in the percentage of Ki67-positive cells in a tumor may be used as an early predictor of treatment efficacy [54]. Despite Ki67 being so widely used as a robust marker for proliferation, its cellular function remains mysteriously unclear. By contrast, the other two markers we chose (PCNA and MCM2) are important participants in the process of DNA replication. In addition to being involved in DNA replication and nucleic acid metabolism, PCNA is also involved in DNA excision repair, cell cycle control, chromatin assembly, and RNA transcription. Since PCNA levels spike during the S and G2/M phases of the cell cycle but is very low in quiescent cells [55], the immunohistochemical staining of PCNA has been extensively used in BC diagnosis and predicting prognosis [55,56]. Increased PCNA expression also correlates with a shorter DFS and OS time in patients with BC [56]. A notable caveat with the use of PCNA is that its expression is not limited to proliferating cells, as PCNA is also involved in DNA damage repair [57,58]. MCM proteins are the key factor for the initiation of DNA replication. In addition, they are required for replication elongation and are implicated in cohesion, condensation, transcription, and recombination of DNA [59]. The family of MCM proteins mainly includes six major proteins, MCM2 through MCM7. Because MCM activity is essential for DNA replication in dividing cells and is lost in quiescence [60], these proteins are also excellent markers for proliferation. Many molecular studies have suggested that increased levels of MCMs may not only be a marker of proliferative malignant cells [61,62] but may also indicate precancerous cells and the potential for recurrence [63,64]. MCM2 is a strong prognostic marker in BC because its high expression is associated with survival, regional recurrence, and distant metastases [65]. Using the bc-Genexminer analysis platform [66], we examined correlations in expression between the 15 MMB-FoxM1-regulated cell cycle genes and the aforementioned established markers of proliferation among TNBC tumors. Our analysis showed a strong statistically significant positive correlation between the expression of all 15 genes and the expression of Ki67, PCNA, and MCM2 among TNBCs (Figure 1). Among BCs, each of these 15 genes was expressed at a statistically significantly higher level in Ki67-high breast tumors (Ki67% > 25%) compared to Ki67-low breast tumors (Supplementary Figure S5A–O), which supported the notion that upregulation of these genes was strongly associated with proliferation among all breast tumors.

Cohen et al. [45] studied cell proliferation in vivo without introducing unwanted biochemical perturbations or relying on transgenic models, and identified global in vivo circuitries that regulate the mammalian cell cycle; the genes they identified were highly expressed in proliferating cells but minimally expressed in resting cells, resulting in a strong enrichment for cell cycle activators. Six out of the 15 genes of our interest—KIF11, KIF4A, KIF2C, KIF20A, CENPF, UBE2C—as well as FOXM1 and ASPM, were identified by Cohen et al., as part of their 83-gene signature of proliferation, which corroborates the idea that the genes we focused on in the context of AR-low TNBC, are involved more generally in supporting high proliferation in a wider variety of tumor types that are particularly proliferative. Grant et al. [67] found 96 genes that were cell cycle-regulated in four distinct cell types (U2OS, HeLa, HaCaT, and foreskin fibroblasts), suggesting that these genes played a critical role in essential biological processes required for successful cell division. These common genes are often bound by E2F1 (for S phase) or FoxM1 (for G2/M phase) at their promoters. Among the genes whose promoters were bound by FoxM1, and wherein FoxM1 binding stimulated gene transcription, were 9 out of the 15 genes we focused on in our study; the nine genes were KIF11, KIF4A, KIF2C, KIF20A, CENPA, CENPF, OIP5, UBE2C, and UBE2S. ASPM was also found to be part of the 96-gene proliferation signature identified by Grant et al. [67], which suggested that these genes may be components of a transcriptomic signature of cell proliferation and may contribute mechanistically to the highly proliferative fraction observed in AR-low TNBC. Thirteen out of the 15 genes we centered our study on—KIF11, KIF14, KIF2C, KIF20A, KIF4A, CENPA, CENPO, CENPL, CENPF, OIP5, UBE2C, UBE2S, and UBE2T—were also identified as MMB-FoxM1 G2/M target genes by Sadasivam et al. [68]. Our study thus identified a group of FoxM1-regulated genes, upregulated in AR-low TNBCs, that are strongly associated with the high proliferation phenotype of AR-low TNBCs. These findings raised the possibility that the overexpression of these genes may be essential to mechanistically support the proliferation needs of AR-low TNBC tumors.

### 2.3. FOXM1-Regulated Mitotic Kinesins Are Overexpressed in AR-Low and TP53-Mutant Breast Tumors and Promote Proliferation and Chromosomal Instability

We showed earlier that expression levels of the five mitotic kinesins (KIF14, KIF11, KIF4A, KIF2C, and KIF20A) downstream of FOXM1 highly correlate with proliferation markers not just in TNBC (Figure 1) but more broadly across breast tumors (Supplementary Figure S5), and these five kinesin motors are overexpressed not only in AR-low TNBC (Supplementary Figures S1, S3 and S4) but also among AR-low breast tumors more broadly (Supplementary Figure S2). Thus, we decided to examine the patterns of expression of these kinesins more closely.

**Figure 2:**
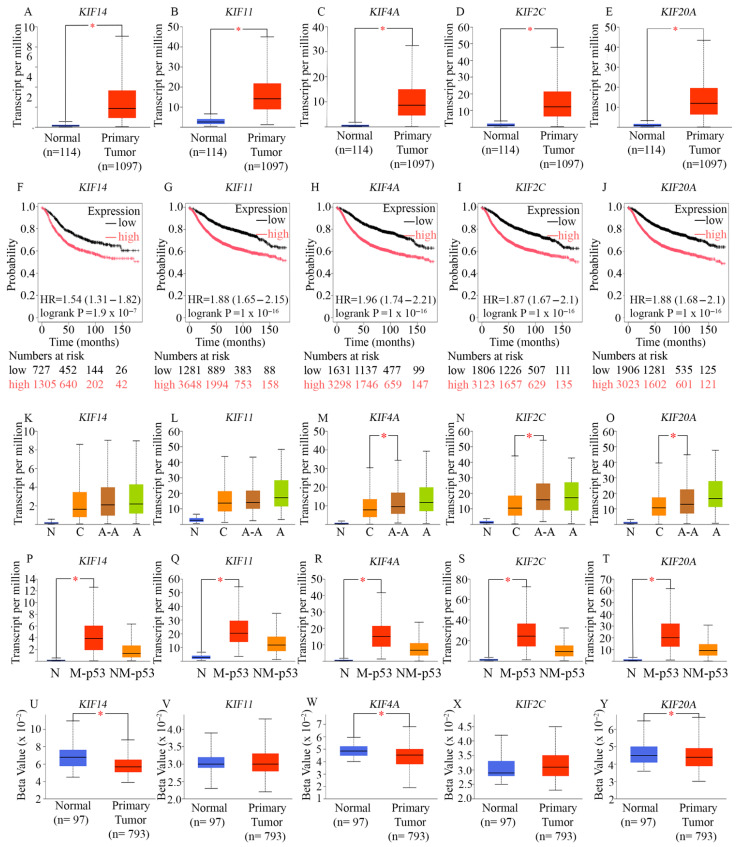
Analysis of the expression levels of genes encoding 5 mitotic kinesins in diverse breast tumors and evaluation of the prognostic significance of their overexpression. (**A**–**E**) Box-whisker plots comparing the expression levels of KIF14 (**A**), KIF11 (**B**), KIF4A (**C**), KIF2C (**D**), and KIF20A (**E**) in primary breast tumor tissues (red boxes) contrasted to normal tissues (blue boxes). The UALCAN platform was used to analyze TCGA level 3 RNA sequencing data. The red asterisk (*) indicates a statistically significant difference (*p* < 0.05). (**F**–**J**) Kaplan–Meier survival analysis evaluating the prognostic significance of the expression of the indicated mitotic kinesins, performed using microarray data from TCGA breast tumors and displayed using the KM Plotter tool. The red line depicts the survival of patients with expression levels of the genes above the cut-point, while the black line represents the recurrence-free survival of patients with expression levels below the cut-point. The analysis was conducted using the KM Plotter’s JetSet optimal microarray probe set and an optimal cutoff for recurrence-free survival over 180 months, without restricting to specific BC subtypes. (**K**–**O**) Analysis of the expression levels of the indicated mitotic kinesin genes in breast tumors from patients of different races (self-identified). “N” represents normal breast tissues with a sample size *n* = 114 for all individuals, “C” represents Caucasians with a sample size *n* = 748, “A-A” represents African Americans with a sample size *n* = 179, and “A” represents Asians with a sample size *n* = 61. Analysis of TCGA RNA sequencing data was performed on the UALCAN platform and visualized using a box-whisker plot. A red asterisk (*) denotes a statistically significant difference in expression between breast tumors from Caucasian and African-American patients (*p* < 0.05). (**P**–**T**) Analysis of the expression levels of the indicated mitotic kinesin genes in breast tumors of differing TP53 mutation status. This analysis used TCGA RNA sequencing data and was performed via the UALCAN platform. The box-whisker plots display the expression of the indicated kinesins in tumors with mutated TP53 (M-p53), contrasting with non-mutated TP53 (NM-p53). A red asterisk (*) denotes a statistically significant difference in expression between the two indicated groups (*p* < 0.05). (**U**–**Y**) Analysis of the promoter methylation profiles of the indicated mitotic kinesin genes in breast tumors. This analysis used TCGA RNA sequencing data and was performed via the UALCAN platform. The box-whisker plots display the methylation level of the promoters of the indicated genes in normal breast tissues (blue boxes) vs. breast invasive carcinoma (red boxes). A red asterisk (*) denotes a statistically significant difference in promoter methylation between the two (*p* < 0.05).

Among BCs, we found significant upregulation of KIF14 (Figure 2A), KIF11 (Figure 2B), KIF4A (Figure 2C), KIF2C (Figure 2D), and KIF20A (Figure 2E) in breast tumor samples of the UALCAN dataset (Figure 2A–E), compared to normal samples. Next, we examined associations between the expressions of KIF14, KIF11, KIF4A, KIF2C, and KIF20A, as well as BC patients’ RFS, using the KM Plotter online tool [69]. We found that high levels of expression of KIF14 (Figure 2F), KIF11 (Figure 2G), KIF4A (Figure 2H), KIF2C (Figure 2I), and KIF20A (Figure 2J) predicted significantly poorer RFS of BC patients, suggesting that upregulation of these mitotic kinesins could potentially contribute to disease progression. Since AR-negative TNBC is more commonly diagnosed among African American women and is believed to underlie the stark racial disparity in BC outcomes in the US [70], we examined the race-wise expression of the mitotic kinesins KIF14 (Figure 2J), KIF11 (Figure 2K), KIF4A (Figure 2L), KIF2C (Figure 2M), and KIF20A (Figure 2N), using data from 1102 race-annotated breast tumors in the UALCAN dataset. We found that KIF4A, KIF2C, and KIF20A showed a significantly higher expression level among African American BC patients compared to Caucasian/White BC patients. Taken together, our analyses suggest that among breast tumors in general, and specifically among AR-low TNBCs, a group of key mitotic kinesins is upregulated, and their upregulation portends poorer outcomes. Furthermore, KIF14 (Figure 2P), KIF11 (Figure 2Q), KIF4A (Figure 2R), KIF2C (Figure 2S), and KIF20A (Figure 2T) were all significantly overexpressed in breast tumors harboring mutant TP53 compared to breast tumors harboring non-mutant TP53. This finding supports our previous data showing that FOXM1 itself was also upregulated in TP53-mutant breast tumors [13]. We then confirmed our findings using the muTarget tool [69] to investigate the effect of mutations in the TP53 coding region (i.e., our input genotype) that have a prevalence of at least 2%, on downstream gene expression in a sample set comprising 305 TP53-mutant and 674 TP53-wild-type BCs found in TCGA. In this dataset, KIF14 showed a 1.96-fold upregulation (p = 1.77 × 10^−38^), KIF11 showed a 1.62-fold upregulation (p = 8.57 × 10^−33^), KIF4A showed a 1.94-fold upregulation (p = 3.89 × 10^−47^), KIF2C showed a 2.48-fold upregulation (p = 4.59 × 10^−62^), KIF20A showed a 1.94-fold upregulation (p = 2.02 × 10^−44^), in TP53-mutant versus TP53-wild-type BCs. These data compellingly indicate that (a) AR-low breast tumors and AR-low TNBCs in particular, (b) Ki67-high BCs, and (c) TP53-mutant breast tumors all show a significant upregulation of KIF14, KIF11, KIF4A, KIF2C, and KIF20A.

CHR elements have previously been identified in the promoters of mitotic kinesins [71]. Chromatin Immunoprecipitation (ChIP) assays performed in BC cell lines confirmed strong binding of B-MYB and FOXM1 to the promoters of KIF14, KIF20A, and KIF4A, and moderate binding to the KIF2C promoter [72]. RNA interference (RNAi) experiments showed that depletion of MMB or FOXM1 subunits significantly inhibited the expression of KIF14, KIF4A, KIF20A, and KIF2C, suggesting that these four mitotic kinesins are direct transcriptional targets of MMB-FoxM1 in BC [72]. KIF11 expression was found to be independent of MMB-FoxM1 [72]. To better understand if under-methylation of promoter DNA contributes to the overexpression of these mitotic kinesins, we utilized the UALCAN promoter methylation analysis tool (Figure 2U–Y). The beta value presented indicates the level of DNA methylation, ranging from 0 (unmethylated) to 1 (fully methylated). Promoter methylation analysis of KIF4A, KIF14, and KIF20A, through the UALCAN database, revealed statistically significant hypomethylation at their promoter regions, indicating potential epigenetic regulation. These findings suggest that, in addition to transcriptional upregulation mediated by factors such as MMB-FoxM1, epigenetic mechanisms may also contribute to the overexpression of KIF14, KIF4A, and KIF20A.

Overexpression of mitotic kinesins in cancer cells likely alters spindle dynamics, chromosome segregation, and cytokinesis, promoting decreased fidelity of chromosome segregation or chromosomal instability (CIN) and aneuploidy, which are linked to tumorigenesis, chemoresistance, and cancer progression. KIF20A and KIF4A are part of the gene expression-based CIN75 chromosomal instability signature that is associated with adverse clinical outcomes in multiple cancers [73]. CIN can spur tumor evolution by resulting in loss of heterozygosity (LOH) of tumor suppressors or by creating imbalances or structural changes that culminate in the overexpression of oncogenes. Aneuploidy (gains/losses of whole chromosomes or large fragments of chromosomes) is prevalent in ~90% of solid tumors [74,75]. Pfister et al. [76] developed a computational method to quantify the degree of aneuploidy or structural rearrangements of large chromosome regions. Shifts in alternate allele frequencies (AAFs) occur as a result of aneuploidy, and these shifts can be assessed using TCGA exome sequencing datasets. In tumors, AAF values reflect a combination of chromosomal copy number in individual cells and the fraction of cells carrying aneuploid genomes. Pfister et al. [76] identified heterozygous single nucleotide polymorphisms (SNPs) and calculated their AAFs for 522 human breast tumors from TCGA. The method measures changes in copy number of both whole chromosomes and large fragments of chromosomes, to yield a metric called functional aneuploidy (FA). The standard deviation of these AAF distributions was used as a robust, assumption-free measure of FA, with broader distributions indicating higher prevalence of aneuploidy. The method successfully segregated breast tumors based on their FA levels—the 100 highest FA tumors had an average of 15.6 chromosomes with LOH events, compared to 0.97 in the 100 lowest FA tumors. This study by Pfister et al. [76] identified two major factors associated with high FA in breast tumors: (i) TP53 mutations and (ii) overexpression of specific mitotic transcriptional regulators. Importantly, overexpression of FOXM1, MYBL2, and E2F1 mRNA correlated with high FA scores across all breast tumor subtypes. Furthermore, the DREAM complex, MMB, and FoxM1/MuvB transcriptional complexes regulate the transcription of 92 of the 100 most overexpressed genes among high-FA breast tumors in TCGA, indicating that the overexpression of these three transcription factors and their downstream targets directly and potently drives high functional aneuploidy. Among the top 100 most overexpressed genes among high-FA breast tumors in TCGA were: KIF14 (ranked 64), KIF4A (ranked 41), KIF2C (ranked 50), KIF20A (ranked 78), FOXM1 (ranked 9), and ASPM (ranked 49). Thus, four out of the five mitotic kinesins we identified as being responsible for transcriptional chaos and high proliferation in AR-low TNBC are also major drivers of functional aneuploidy in this high-risk, high-need BC subtype, presumably because overexpression of KIFs leads to an increased frequency of lagging chromosomes, and thus, the generation and propagation of functional aneuploidy. These data corroborate the CIN75 study [73] and greatly extend the idea that aneuploidy (and, likely, CIN) in BC more broadly, and AR-low TNBC in particular, is associated with, and likely, causally related to the overexpression of mitotic kinesins.

Our data showed that overexpression of KIF14, KIF11, KIF4A, KIF2C, and KIF20A was associated with mutations in TP53, and Pfister et al. [75] found that TP53 mutations were highly enriched in the high FA tumors. TP53 mutations have previously been connected to aneuploidy in tumors [77,78]. Experimental evidence in *Xenopus* embryos suggests that TP53 mutations do not directly lower the fidelity of mitosis; instead, TP53’s role as the guardian of ploidy prevents the survival and proliferation of cells that (i) have undergone chromosomal mis-segregation, or (ii) harbor chromosome fragments, by inducing cell cycle arrest, apoptosis, or entosis [76,79]. TP53 loss of function thus leads to the survival and persistence of aneuploid cells, leading to an increased level of functional aneuploidy. To demonstrate causation, Pfister et al. [76] injected mRNA encoding human MYBL2, E2F1, and FOXM1 into *Xenopus laevis* embryos. This overexpression was sufficient to increase the rate of lagging anaphase chromosomes in this non-transformed vertebrate tissue and provided evidence for a direct causal link between the overexpression of these factors and reduced mitotic fidelity, and the generation of a significantly higher percentage of micronuclei, which often form from lagging chromosomes. In fact, the study found a strong co-association of every combination of TP53 mutations and the overexpression of MYBL2 and FOXM1. Cellular senescence is one of the intrinsic safeguards against cancer progression. Although different triggers induce cellular senescence, p53 is well documented to play a critical role in its induction. Kif2C has been reported to play an important role in the regulation of cellular senescence in human cells through a p53-dependent pathway [80]; TP53 loss of function would presumably compromise this induction of senescence, leading to the persistence of cells with mitotic errors. Taken together, these lines of evidence converge on the idea that overexpression of mitotic kinesins strongly promotes proliferation in AR-low TNBCs and TP53-mutant breast tumors, although the proliferation involves erroneous chromosome segregation and generation of extensive CIN and aneuploidy.

### 2.4. FOXM1-Regulated Centromeric Proteins Are Overexpressed in AR-Low and TP53-Mutant Breast Tumors and Promote Proliferation and Poor Survival

Earlier, we showed that expression levels of the five centromeric proteins (CENPA, CENPO, CENPL, CENPF, and OIP5) we identified highly correlate with expression of proliferation markers, not just in TNBC (Figure 1) but more broadly across breast tumors (Supplementary Figure S5), and these five centromeric proteins are overexpressed not only in AR-low TNBC (Supplementary Figures S1, S3 and S5) but also among AR-low breast tumors (Supplementary Figure S2). Therefore, we evaluated their patterns of expression more closely.

**Figure 3:**
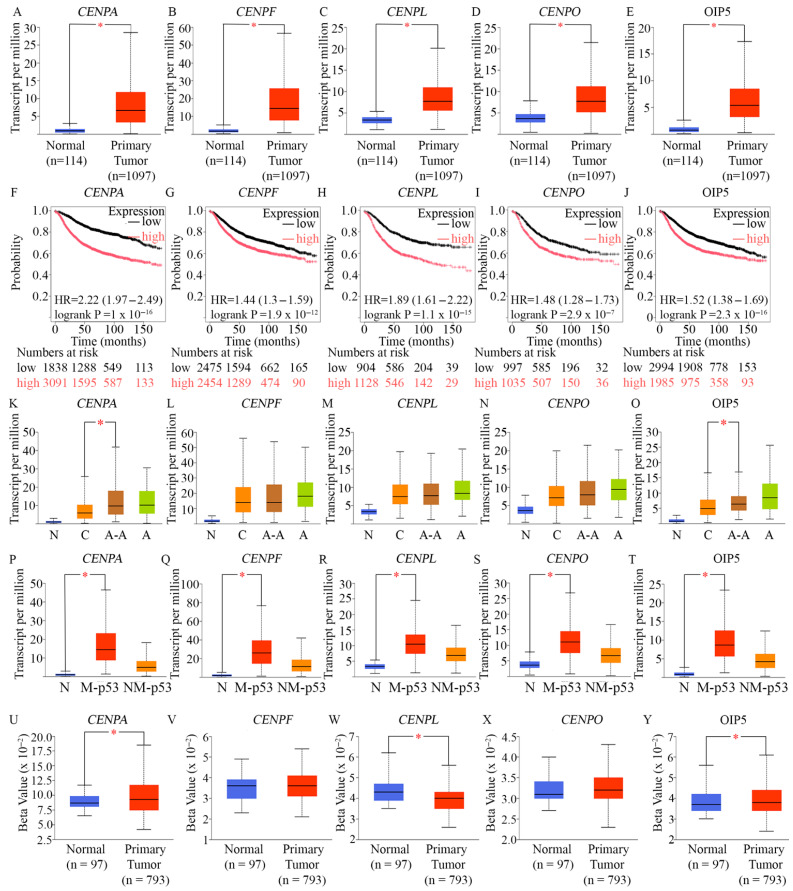
Analysis of the expression levels of genes encoding 5 centromeric proteins in diverse breast tumors and evaluation of the prognostic significance of their overexpression. (**A**–**E**) Box-whisker plots compare the expression levels of CENPA (**A**), CENPF (**B**), CENPL (**C**), CENPO (**D**), and OIP5 (**E**) in primary breast tumor tissues (red boxes) contrasted to normal tissues (blue boxes). The UALCAN platform was used to analyze TCGA level 3 RNA sequencing data. The red asterisk (*) indicates a statistically significant difference (*p* < 0.05). (**F**–**J**) Kaplan–Meier survival analysis evaluating the prognostic significance of the expression of the indicated centromeric proteins, performed using microarray data from TCGA breast tumors and displayed using the KM Plotter tool. The red line depicts the recurrence-free survival of patients with expression levels of the genes above the cut-point, while the black line represents the recurrence-free survival of patients with expression levels below the cut-point. The analysis was conducted using the KM Plotter’s JetSet optimal microarray probe set and an optimal cutoff for recurrence-free survival over 180 months, without restricting to specific BC subtypes. (**K**–**O**) Analysis of the expression levels of the indicated centromeric genes in breast tumors from patients of different races (self-identified). “N” represents normal breast tissues with a sample size *n* = 114 for all individuals, “C” represents Caucasians with a sample size *n* = 748, “A-A” represents African Americans with a sample size *n* = 179, and “A” represents Asians with a sample size *n* = 61. Analysis of TCGA RNA sequencing data was performed on the UALCAN platform and visualized using a box-whisker plot. A red asterisk (*) denotes a statistically significant difference in expression between breast tumors from Caucasian and African-American patients (*p* < 0.05). (**P**–**T**) Analysis of the expression levels of the indicated centromeric genes in breast tumors of differing TP53 mutation status. This analysis used TCGA RNA sequencing data and was performed via the UALCAN platform. The box-whisker plots display the expression of the indicated kinesins in tumors with mutated TP53 (M-p53), contrasting with non-mutated TP53 (NM-p53). A red asterisk (*) denotes a statistically significant difference in expression between the two indicated groups (*p* < 0.05). (**U**–**Y**) Analysis of the promoter methylation profiles of the indicated centromeric genes in breast tumors. This analysis used TCGA RNA sequencing data and was performed via the UALCAN platform. The box-whisker plots display the methylation level of the promoters of the indicated genes in normal breast tissues (blue boxes) vs. breast invasive carcinoma (red boxes). A red asterisk (*) denotes a statistically significant difference in promoter methylation between the two (*p* < 0.05).

In BC samples from the UALCAN dataset, we observed marked overexpression of the five centromeric proteins—CENPA, CENPO, CENPL, CENPF, and OIP5—compared to normal breast tissue (Figure 3A–E). To assess the prognostic relevance of these genes, we performed separate Cox proportional hazards regression analyses using the Kaplan–Meier Plotter [69]. High expression levels of each centromeric gene were consistently associated with significantly reduced RFS in BC patients, as visualized by Kaplan–Meier curves (Figure 3F–J). Given the association of AR-negative TNBC with poorer prognosis in African American women [70], we next assessed racial disparities in expression using race-annotated data from 1102 tumors in the UALCAN dataset. CENPA and OIP5 were expressed at significantly higher levels in African American patients compared to their Caucasian/White counterparts (Figure 3K–O). Collectively, these findings indicate that these centromeric proteins are frequently upregulated in BCs—particularly in AR-low TNBCs—and that their elevated expression correlates with worse clinical outcomes.

We further investigated whether expression of these centromeric proteins was influenced by the TP53 mutational status of breast tumors. Our analysis of TCGA RNA-seq data through the UALCAN revealed that CENPA (Figure 3P), CENPF (Figure 3Q), CENPL (Figure 3R), CENPO (Figure 3S), and OIP5 (Figure 3T) were all significantly upregulated in TP53-mutant tumors compared to those with wild-type TP53, consistent with previous findings on their upstream regulator, FOXM1 overexpression in TP53-mutant BCs [13]. To corroborate this, we utilized the muTarget platform [69] to analyze the impact of TP53 mutations (≥2% prevalence) on gene expression in a TCGA cohort of 305 mutant and 674 wild-type samples. The results confirmed substantial upregulation of the centromeric proteins in TP53-mutant tumors: CENPA (2.88-fold; p = 6.91 × 10^−67^), CENPO (1.62-fold; p = 1.11 × 10^−42)^, CENPL (1.52-fold; p = 3.75 × 10^−34^), CENPF (1.86-fold; p = 2.29 × 10^−38^), and OIP5 (1.81-fold; p = 2.13 × 10^−44^). In cells with functional p53, defects in chromosome segregation normally activate p53-mediated responses, including cellular senescence induction. When a cell harbors p53 loss-of-function mutations, the unchecked entry into mitosis of cells whose centromeres cannot support proper chromosome segregation causes the generation and persistence of aneuploid cells [81]. New studies have found that a reduction in CENPA expression can prompt a p53-dependent cellular senescence response [82]. This response presumably prevents centromere-defective cells from proceeding through mitosis—a process that could potentially lead to the generation of aneuploid cells [83]. Another study also identified p53 as a key determinant of how CENPA impacts cell state, cell identity, and therapeutic response [84]. If p53 is functional, CENPA overexpression promotes senescence and radiosensitivity. By contrast, when p53 is inactivated, CENPA overexpression promotes epithelial-to-mesenchymal transition (EMT)—a precursor for metastasis. Thus, CENPA overexpression has an unanticipated function in promoting cell fate reprogramming, with important implications for development and tumor evolution [84]. Evidence now shows that TP53 binds to the CENPA promoter and directly represses CENPA expression [82]. CENPA is deposited in a replication-independent manner by a dedicated histone chaperone, HJURP (Holliday junction recognition protein), to replace its canonical counterpart. TP53 normally binds to the HJURP promoter and represses its transcription. In a TP53-deficient background, once HJURP or CENPA is upregulated at the protein level, it can, in turn, stabilize the other by protecting it from proteasomal degradation [82]. As a result, CENPA overexpression is accompanied by HJURP overexpression in TP53-deficient contexts, leading to CENPA misincorporation into non-centromeric regions of chromosomes and propagation of CIN [82].

To explore whether reduced promoter DNA methylation may be linked to the elevated expression of these centromeric proteins, we conducted promoter methylation analysis using the UALCAN platform (Figure 3U–Y). Analysis of CENPA, CENPL, and OIP5 genes revealed significantly lower methylation levels at their promoter regions in breast tumor samples, consistent with promoter hypomethylation. These observations suggest that, in addition to transcriptional hyperactivation by regulatory complexes such as MMB–FOXM1, epigenetic deregulation may also contribute to the aberrant overexpression of these centromeric proteins. Taken together, these analyses suggest that AR-low BCs, particularly AR-low TNBCs, as well as Ki67-high and TP53-mutant breast tumors, are characterized by co-upregulation of CENPA, CENPO, CENPL, CENPF, and OIP5—highlighting their potential contributions to disease progression and poor prognosis.

### 2.5. Regulators of Protein Degradation Play Prominent Roles in Driving Poor Outcomes in AR-Low TNBC and TP53-Mutant Breast Tumors

Earlier, we identified five proteins that play key roles in ubiquitin (Ub)-related protein degradation and aggrephagy—UBE2S, UBE2C, UBE2T, PSMD14, and TUBA1B—that are significantly overexpressed in AR-low TNBC. The expression levels of these genes correlate strongly with proliferation markers not only in TNBC (Figure 1) but across breast tumors more broadly (Supplementary Figure S5). Moreover, these proteins are elevated in both AR-low TNBC (Supplementary Figures S1, S3 and S4) and in AR-low BCs overall (Supplementary Figure S2). These indications prompted a more detailed examination of their expression profiles.

**Figure 4:**
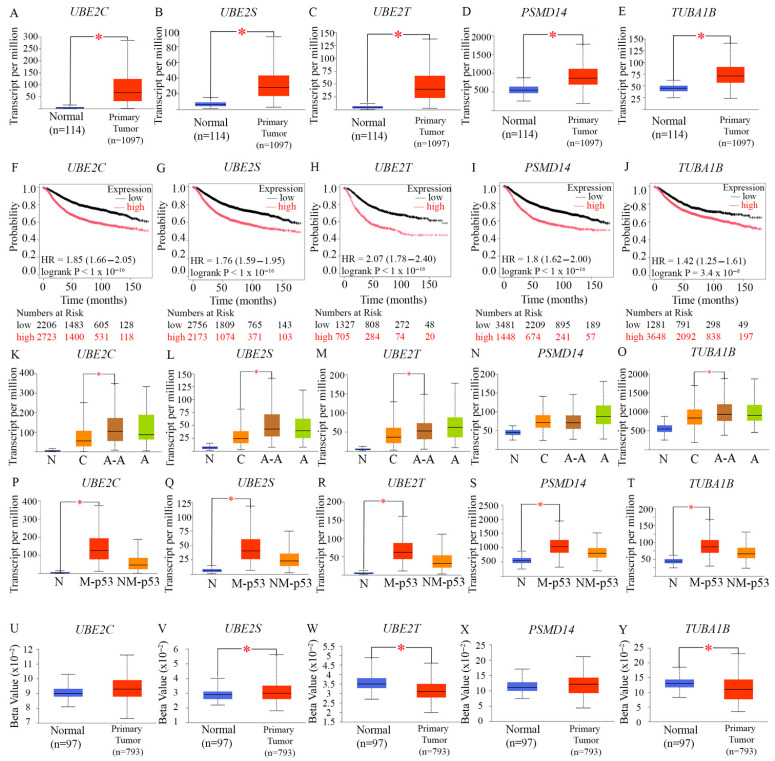
Analysis of the expression levels of genes encoding 5 proteolysis-regulatory proteins in diverse breast tumors and evaluation of the prognostic significance of their overexpression. (**A**–**E**) Box-whisker plots compare the expression levels of UBE2C (**A**), UBE2S (**B**), UBE2T (**C**), PSMD14 (**D**), and TUBA1B (**E**) in primary breast tumor tissues (red boxes) contrasted to normal tissues (blue boxes). The UALCAN platform was used to analyze TCGA level 3 RNA sequencing data. The red asterisk (*) indicates a statistically significant difference (*p* < 0.05). (**F**–**J**) Kaplan–Meier survival analysis evaluating the prognostic significance of the expression of the indicated proteolysis-regulatory proteins, performed using microarray data from TCGA breast tumors and displayed using the KM Plotter tool. The red line depicts the recurrence-free survival of patients with expression levels of the genes above the cut-point, while the black line represents the recurrence-free survival of patients with expression levels below the cut-point. The analysis was conducted using the KM Plotter’s JetSet optimal microarray probe set and an optimal cutoff for recurrence-free survival over 180 months, without restricting to specific BC subtypes. (**K**–**O**) Analysis of the expression levels of the indicated proteolysis-regulatory genes in breast tumors from patients of different races (self-identified). “N” represents normal breast tissues with a sample size *n* = 114 for all individuals, “C” represents Caucasians with a sample size *n* = 748, “A-A” represents African Americans with a sample size *n* = 179, and “A” represents Asians with a sample size *n* = 61. Analysis of TCGA RNA sequencing data was performed on the UALCAN platform and visualized using a box-whisker plot. A red asterisk (*) denotes a statistically significant difference in expression between breast tumors from Caucasian and African-American patients (*p* < 0.05). (**P**–**T**) Analysis of the expression levels of the indicated proteolysis-regulatory genes in breast tumors of differing TP53 mutation status. This analysis used TCGA RNA sequencing data and was performed via the UALCAN platform. The box-whisker plots display the expression of the indicated kinesins in tumors with mutated TP53 (M-p53), contrasting with non-mutated TP53 (NM-p53). A red asterisk (*) denotes a statistically significant difference in expression between the two indicated groups (*p* < 0.05). (**U**–**Y**) Analysis of the promoter methylation profiles of the indicated proteolysis-regulatory genes in breast tumors. This analysis used TCGA RNA sequencing data and was performed via the UALCAN platform. The box-whisker plots display the methylation level of the promoters of the indicated genes in normal breast tissues (blue boxes) vs. breast invasive carcinoma (red boxes). A red asterisk (*) denotes a statistically significant difference in promoter methylation between the two (*p* < 0.05).

Data from the UALCAN dataset showed that UBE2C (Figure 4A), UBE2S (Figure 4B), UBE2T (Figure 4C), PSMD14 (Figure 4D), and TUBA1B (Figure 4E) were all significantly upregulated in breast tumor samples relative to normal tissue. To determine the prognostic relevance of this pattern of overexpression, we evaluated associations between gene overexpression and RFS for each gene of interest individually using Cox proportional hazards models in the Kaplan–Meier Plotter tool. Survival curves demonstrated that elevated expression of each of these five genes of interest (Figure 4F–J) was associated with a significantly shorter recurrence-free interval, suggesting roles in disease progression. We also compared the expression levels of these genes in BC patients categorized by self-declared race in 1102 annotated breast tumor samples from UALCAN. The expression levels of UBE2C, UBE2S, UBE2T, and TUBA1B were notably higher in African American patients than in Caucasian/White patients. Collectively, these findings indicate that in BC—particularly in AR-low TNBC—a subset of proteins that play key roles in protein degradation and cell cycle progression is consistently overexpressed, with higher expression correlating with worse outcomes. Further analysis revealed that all five proteins—UBE2C (Figure 4P), UBE2S (Figure 4Q), UBE2T (Figure 4R), PSMD14 (Figure 4S), and TUBA1b (Figure 4T)—were significantly elevated in TP53-mutant tumors compared with TP53–wild-type tumors, consistent with prior results showing FOXM1 overexpression in the TP53-mutant setting [13]. These observations were validated using the muTarget platform, which compared gene expression patterns in 305 TP53-mutant and 674 TP53-wild-type BCs from TCGA. The TP53-mutant group exhibited substantial increases in UBE2C (2.30-fold, p = 1.76 × 10^−48^), UBE2S (1.69-fold, p = 5.60 × 10^−27^), UBE2T (1.61-fold, p = 4.72 × 10^−30^). No data was available for analysis of PSMD14 and TUBA1B levels in this platform. Together, these data strongly support that AR-low BCs (including AR-low TNBC), Ki67-high tumors, and TP53-mutated breast tumors share a common phenotype of elevated expression of these proteolysis-regulatory proteins. To assess whether reduced promoter methylation contributes to the elevated expression of these proteolysis-related genes, we employed the UALCAN promoter methylation analysis platform (Figure 4U–Y). Results showed significant promoter hypomethylation for UBE2T and TUBA1B in breast tumors, suggesting that epigenetic mechanisms may contribute to the overexpression of these two genes in addition to transcriptional activation driven by regulators such as the MMB–FOXM1 complex. Collectively, these findings indicate that AR-low BCs—especially AR-low TNBC—as well as Ki67-high and TP53-mutant breast tumors, exhibit a coordinated overexpression of UBE2C, UBE2S, UBE2T, PSMD14, and TUBA1B, underscoring their likely roles in driving tumor progression and unfavorable clinical outcomes, via a dysregulation of proteolysis pathways.

### 2.6. Overexpression of the 15-Gene Set Associated with Proliferation and Genomic Instability Is Associated with a Characteristic Tumor Microenvironment in BC

**Figure 5:**
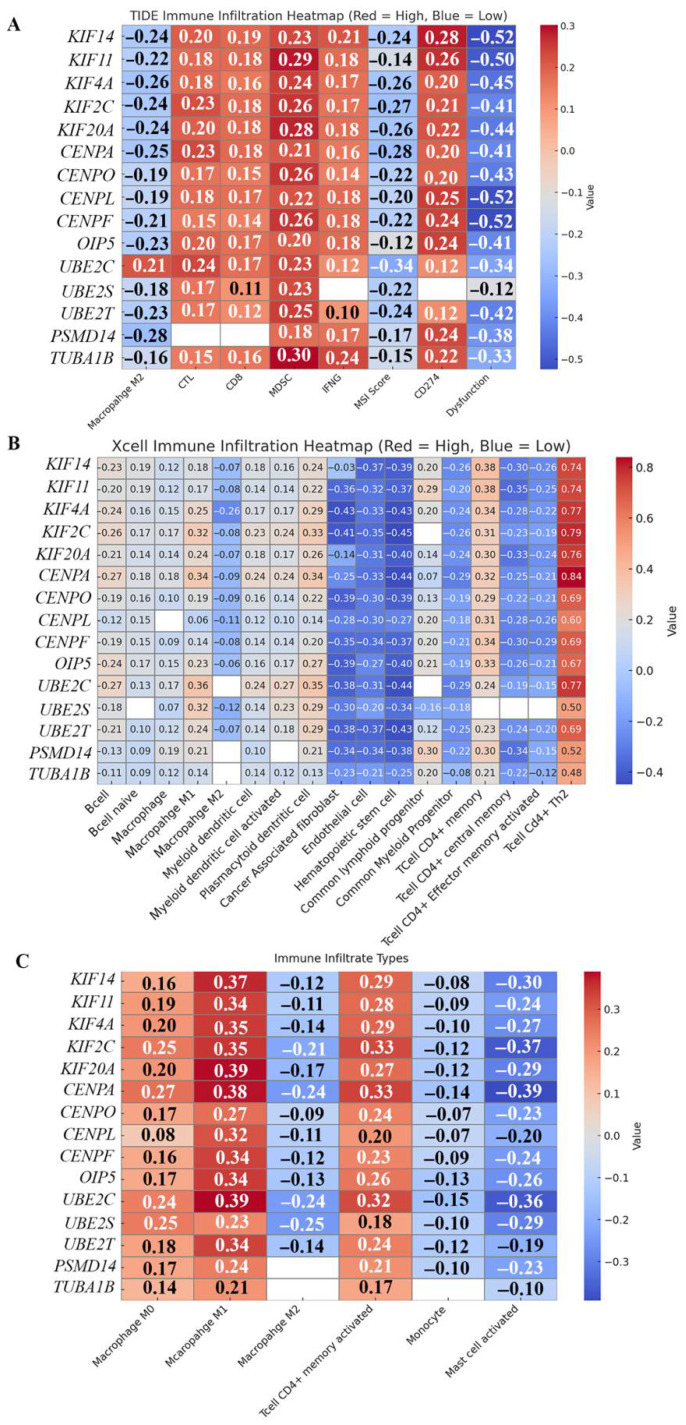
Tumor microenvironmental features associated with the 15-Gene set in BC. (**A**) Spearman correlations between expression of the 15-gene cell cycle regulatory gene set and immune cell infiltration, estimated using the TIDE algorithm via the TIMER 2.0 platform. Only statistically significant pairwise correlations are depicted (*p* < 0.05). Statistically significant negative correlations are depicted in shades of blue, and statistically significant positive correlations are depicted in shades of red. White boxes represent the absence of a statistically significant correlation. (**B**) Spearman correlations between expression of the 15-gene cell cycle regulatory gene set and immune cell infiltration, estimated using the XCELL algorithm via the TIMER 2.0 platform. Only statistically significant pairwise correlations are depicted (*p* < 0.05). Statistically significant negative correlations are depicted in shades of blue, and statistically significant positive correlations are depicted in shades of red. White boxes represent the absence of a statistically significant correlation. (**C**) Spearman correlations between expression of the 15-gene cell cycle regulatory gene set and immune cell infiltration, estimated using the CIBERSORT algorithm via the TIMER 2.0 platform. Only statistically significant pairwise correlations are depicted (*p* < 0.05). Statistically significant negative correlations are depicted in shades of blue, and statistically significant positive correlations are depicted in shades of red. White boxes represent the absence of a statistically significant correlation.

Characterizing the influence of actionable signaling networks on the immune landscape of the tumor microenvironment (TME) is essential for understanding in vivo response to therapeutic intervention. To gain insights into the composition of the TME in breast tumors that overexpress the FOXM1-regulated 15 gene-set, three complementary deconvolution algorithms—TIDE, XCell, and CIBERSORT—were leveraged via the TIMER 3.0 web platform (http://timer.cistrome.org/, last accessed on 13 July 2025). We examined the Spearman correlation between the expression of the 15 cell cycle regulators downstream of FOXM1 at the core of our study, and the infiltration landscape of the TME in BC [85,86] (Figure 5A–C). Despite methodological differences among the algorithms, several convergent patterns emerged, underscoring a consistent pattern of association between gene expression and TME composition.

The Tumor Immune Dysfunction and Exclusion (TIDE) algorithm [87] models two key mechanisms of tumor immune evasion: (i) T cell dysfunction, by detecting prognostic gene expression-based signatures indicative of loss of T cell effector functions within a high cytotoxic T lymphocyte (CTL) tumor environment, and (ii) T cell exclusion, by identifying expression profiles of cells that suppress T cell infiltration, such as cancer-associated fibroblasts (CAFs), myeloid-derived suppressor cells (MDSCs), and M2 (tumor-associated) macrophages. TIDE analysis (Figure 5A) revealed that expression of the 15-gene set was positively correlated with CTLs, MDSCs, interferon-γ (IFNG), and CD274 (PD-L1). Expression of the 15 genes was negatively correlated with M2 macrophages, microsatellite instability (MSI) score, and immune dysfunction score. We found no statistically significant association between the expression of the 15 genes and the Immune Evasion Score. CTLs play a crucial role in directly attacking and eliminating cancer cells [88]; however, exhaustion and dysfunction can occur during cancer progression due to immunosuppression within the TME. CAFs, alternatively activated M2 macrophages, and regulatory T cells can constitute serious immunologic barriers against CTL antitumor immune responses, eventually resulting in CTLs’ exhaustion. A higher number of CD8^+^ CTLs is generally indicative of a favorable response to neoadjuvant therapy in various BC subtypes [89]. Furthermore, studies have revealed that a higher percentage of CD8^+^ T cells is recruited in the tumor of African American BC patients compared to their White counterparts, which is suggestive of the mounting of a strong T-cell-mediated immune response in African American patients [90]. MDSCs represent a diverse population of immune cells that mediate tumor-associated immunosuppression. Within the TME, their principal function is to inhibit T-cell activity, either through antigen-dependent mechanisms or via non-specific pathways. Therefore, MDSCs serve as key drivers of T-cell dysfunction and exhaustion [91]. CD274, also known as PD-L1 (programmed cell death-ligand 1), is a transmembrane protein that interacts with the PD-1 (programmed cell death protein 1) receptor on T cells. This interaction inhibits T cell activation and cytokine production. CD274 expression on tumor cells can help them evade the immune system by suppressing T cell activity, making the PD-1—PD-L1 interaction a target for cancer immunotherapy. Interferon-gamma (IFNG or IFN-γ) is a pivotal cytokine in the TME with dual roles in anti-tumor immunity and immune regulation. Produced mainly by activated CD8^+^ CTLs, NK cells, and Th1 CD4^+^ T cells, IFN-γ enhances tumor antigen recognition by upregulating MHC class I and II expression on tumor and antigen-presenting cells, and it activates macrophages to exert cytotoxic effects against tumor cells [92,93]. However, chronic or excessive IFN-γ signaling can paradoxically promote immune escape by inducing immune checkpoint molecules such as PD-L1 on tumor and stromal cells [94,95,96]. Thus, IFN-γ functions as a double-edged sword—bolstering tumor clearance while simultaneously fostering adaptive resistance mechanisms. IFNG expression and CD274 (PD-L1) are both markers of an inflamed TME. Expression of our 15-gene set was further associated with increased levels of MDSCs, suggesting that the inflammatory context may be counterbalanced by the recruitment of immunosuppressive myeloid populations. Conversely, expression of our 15-gene set demonstrated a negative correlation with M2 macrophages, as well as with both the MSI score and the immune dysfunction score. A low TIDE dysfunction score suggests that the tumor microenvironment may be more amenable to immune activity, possibly with less T-cell exhaustion. However, this alone does not guarantee a good prognosis. Cancer prognosis is influenced by a complex interplay of tumor biology, host factors, and treatment effectiveness. Tumor intrinsic mutations can drive rapid growth, metastasis, or resistance to treatments, regardless of the immune response. A negative correlation between the expression of our 15 genes and the TIDE dysfunction score indicates a potentially more favorable immune environment, suggesting better chances of response to immunotherapy. These results also suggest that the TME of AR-low/TP53-mutant breast tumors expressing this gene program differs from the type of TME typically associated with immune-excluded cancers. Overall, expression of the proliferative 15-gene set correlates with features of both immune activation (CD8^+^ T cells, IFNG, and PD-L1) and immune suppression (MDSCs), while showing inverse associations with M2 macrophages and immune dysfunction. The strength and direction of these correlations reinforce the notion that breast tumors expressing high levels of mitotic kinesins, centromeric proteins, and proteolysis regulators may attract effector T cells, but they also engage immunosuppressive myeloid populations and likely exploit immune checkpoint pathways.

We next interrogated the relationship between the 15-gene set of interest and immune cell composition using the XCELL algorithm (Figure 5B). XCELL is an R-based program that provides a more comprehensive view of the complex cellular landscape, using pre-defined gene signatures to assess the enrichment of each cell type [97]. Macrophages in the TME play a complex and multifaceted role, influencing tumor growth, progression, and response to therapy [98]. Our analysis revealed a positive correlation with innate and antigen-presenting immune subsets, including total macrophages, M1 macrophages, and myeloid dendritic cells (both total and activated). By contrast, expression of the 15-gene set demonstrated negative correlations with M2 macrophages. M1 macrophages are generally associated with anti-tumor functions. They secrete pro-inflammatory cytokines like IL-12 and TNF-α, and chemokines, such as CXCL9, CXCL10, and CXCL11, which can stimulate other immune cells like CD8^+^ T cells and NK cells to kill tumor cells. They also directly mediate tumor cell cytotoxicity and antibody-dependent cell-mediated cytotoxicity. M1 macrophages can inhibit cell proliferation and cause tissue damage through the secretion of pro-inflammatory cytokines and nitric oxide. Some studies, like one focusing on transcriptomically defined M1 macrophages in BC [99], did not find an association between high M1 levels and favorable survival or response to chemotherapy. It appears that the balance of M1 to M2 macrophages, as well as the overall context of the TME, contributes to the ultimate impact on the tumor’s progression. In addition, we observed significant positive correlations with common lymphoid progenitors, CD4^+^ memory T cells, and CD4^+^ Th2 cells, suggesting that tumors expressing these 15 genes are enriched for cell types associated with pro-inflammatory activity and adaptive immune priming. It was also inversely associated with several other immune and stromal compartments, including B cells, CAFs, endothelial cells, hematopoietic stem cells, common myeloid progenitors, CD4^+^ central memory T cells, and CD4^+^ effector–memory-activated T cells. Negative correlations with endothelial cell abundance further implicate these tumors in fostering a TME that is not conducive to angiogenesis. These negative associations indicate that expression of the proliferative gene program coincides with reduced B cell infiltration, lower stromal support, and depletion of certain memory T cell subsets, potentially altering the quality and persistence of immune responses in the TME. Together, these XCELL-derived correlations suggest that tumors with high expression of the 15-gene signature foster a distinct immune milieu, characterized by M1 macrophage and dendritic cell predominance, enrichment of CD4^+^ subsets, but reduced stromal and endothelial support, as well as an absence of immunosuppressive M2 macrophage infiltration.

CIBERSORT analysis (Figure 5C) yielded trends consistent with those observed with TIDE and XCELL: expression of the 15 genes was positively correlated with M0 and M1 macrophages, and CD4^+^ activated memory T cells, but negatively correlated with M2 macrophages, monocytes, and activated mast cells. These findings emphasize a skewing of the myeloid compartment toward pro-inflammatory macrophages, coupled with reduced representation of regulatory and tolerogenic subsets. Taken together, the results across all three algorithms converge on a model in which high expression of mitotic kinesins, centromeric proteins, and proteolysis regulators in AR-low TNBC and p53-deficient breast tumors is associated with a TME enriched for effector and pro-inflammatory immune subsets, but with simultaneous evidence of immune evasion pathways (MDSC infiltration, PD-L1 upregulation, and loss of immune-stimulatory diversity). This duality may help explain how AR-low TNBC and TP53-mutant tumors maintain aggressive proliferation despite the presence of immune activity, underscoring both the therapeutic vulnerabilities and the adaptive complexity of their TME.

## 3. Discussion

The study presented herein explored the patterns of gene overexpression and identified a dysregulated axis that is associated with and likely mechanistically supports the transcriptional chaos, high proliferation, and high genomic instability phenotypes of AR-low TNBC tumors. In doing so, this study moves beyond descriptive subtyping of TNBC to identify mechanistic drivers of the aggressive biology that underlies poor outcomes in AR-low disease. AR-low TNBCs are disproportionately associated with rapid proliferation, chromosomal instability, therapy resistance, and early relapse, yet clinicians currently lack biomarkers that explain why these tumors behave so aggressively or how to intervene effectively. By defining a coherent set of overexpressed cell-cycle and mitotic regulators that support transcriptional chaos and genomic instability, this work provides a biologically grounded framework for risk stratification and therapeutic targeting in a patient population with few actionable options. Importantly, the genes and regulatory circuits identified here are not merely correlated with poor prognosis but are plausibly causal in sustaining hyperproliferation and error-prone mitoses. This opens new avenues for clinical translation, including the development of biomarkers to identify high-risk AR-low tumors, rational combination therapies that exploit vulnerabilities in mitotic control or proteostasis pathways, and strategies to overcome resistance driven by genomic instability. In a disease where standard chemotherapy remains the mainstay of treatment, elucidating the molecular architecture that fuels tumor evolution and treatment failure has direct clinical implications for improving patient outcomes.

Mitotic kinesins are ATP-dependent motor proteins crucial for intracellular transport, mitotic spindle formation and function, chromosome segregation, supernumerary centrosome clustering, and cytokinesis [100,101,102,103]. Overexpression of mitotic kinesins is commonly observed in tumor cells and is implicated in oncogenesis, chemoresistance, and is associated with more advanced disease stages [102,104,105,106,107,108,109]. Our analysis identified five mitotic kinesins (KIF14, KIF11, KIF4A, KIF2C, and KIF20A), upregulated by FOXM1, among a set of core genes responsible for transcriptional chaos in TNBC, that were (a) overexpressed in AR-low TNBC and AR-low BC, and (b) showed a pattern of expression highly associated with proliferation in AR-low TNBC and AR-low breast tumors. Four out of the five mitotic kinesins we identified were ranked among the top 250 genes overexpressed among TNBCs: KIF4A was ranked 10th, KIF2C was ranked 24th, KIF20A was ranked 45th, and KIF11 was ranked 117th [110]. These extremely high rankings suggest the high importance of these kinesins in driving the tumor biology of breast tumors with TN status. A thorough description of the main mitotic functions helmed by these kinesins with a perspective to better understanding their roles in spurring aggressive tumor biology in AR-low TNBC can be found in Supplementary Table S1. Citations [111,112,113,114,115,116,117,118,119,120,121,122,123,124,125,126,127,128,129,130,131,132,133,134,135,136,137,138,139,140,141,142,143,144,145,146,147,148,149] are found in Supplementary Table S1. As summarized in Supplementary Table S1, KIF20A, KIF14, KIF11, KIF4A and KIF2C all have critical functions necessary for precise spindle organization, regulation of microtubule dynamics, chromosome segregation, cleavage furrow formation and maintenance, and cytokinesis. All the five mitotic kinesins we identified were categorized as components of a “12-gene Mitotic kinesin signature (MKS)” and high expression of MKS genes was correlated with worse RFS, OS and DMFS in BC patients [72], suggesting a strong association between overexpression of these kinesins and aggressive BC phenotypes. Their co-overexpression drives proliferation and genomic instability which promotes metastasis, stem cell phenotypes, therapy resistance, and poor survival, making them compelling prognostic biomarkers and therapeutic targets in AR-low BCs.

In addition to the five mitotic kinesins described above, our analysis identified five centromeric proteins (CENPA, CENPO, CENPL, CENPF, and OIP5), upregulated by FOXM1, among the set of core genes responsible for transcriptional chaos in TNBC. Centromeric proteins play prominent roles in establishing centromere identity and function, enabling kinetochore assembly, and ensuring accurate chromosome segregation during mitosis. These identified centromeric proteins were also (a) overexpressed in AR-low TNBC and AR-low BC, and (b) showed a pattern of expression positively associated with high proliferation in AR-low TNBC and AR-low breast tumors. All five centromeric proteins are established transcriptional targets of the MMB-DREAM complex. Four out of these five centromeric genes are also ranked among the highest in the UALCAN TCGA database ranking of genes most overexpressed in TNBC, with CENPA ranked 29th most overexpressed in all TNBCs, CENPF 56th, CENPL 220th, and CENPO 239th. Again, these high rankings suggested that upregulation of these genes likely plays a critical role in the tumor biology of TNBCs. Their known cellular functions within human centromeres and a description of how overexpression of these genes may undergird and power the aggressive clinical behavior of AR-low TNBCs are presented in Supplementary Table S2 (which also includes citations [150,151,152,153,154,155,156,157,158,159,160,161,162,163,164,165,166,167,168,169,170,171,172,173,174,175,176,177,178,179,180,181,182,183,184,185,186,187,188,189,190,191,192,193,194,195,196,197,198,199,200,201,202,203,204,205,206,207,208,209,210,211,212,213,214]). Therefore, the above-described centromeric proteins play pivotal roles in centromere identity establishment and maintenance, kinetochore assembly, formation of high-tension kinetochore-spindle microtubule attachments, proper chromosome alignment at the metaphase plate, and accurate chromosome segregation during anaphase, so that mitosis is error-free. Centromere protein deregulation leads to CIN, a cancer hallmark. Among the five centromeric proteins discussed above, CENPA and CENPO were ranked 34th and 27th, respectively, among the top 100 most overexpressed genes among high-functional aneuploidy breast tumors in TCGA [76]. The CIN75 signature includes OIP5 [73]. CENPF overexpression was significantly associated with markers of CIN, including cyclin E, increased telomerase activity, c-Myc amplification, and aneuploidy [215]. Taken together, dysregulation of the above-mentioned five centromeric proteins favors rapid and uncontrolled proliferation, increased CIN and aneuploidy, and increased chemoresistance, and is correlated with advanced disease stage and poor outcomes in multiple cancer types, including BC; these findings highlight their dual potential as prognostic biomarkers and therapeutic targets.

In addition to the five mitotic kinesins and five centromeric proteins described in previous sections, our research also identified five genes that play vital roles in the process of protein degradation (UBE2S, UBE2C, UBE2T, PSMD14, and TUBA1B), upregulated by FOXM1, among the set of core genes responsible for transcriptional chaos in TNBC. Ubiquitin-dependent proteolysis and aggrephagy modulate the stability and activities of cell cycle regulators, promote fidelity of chromosome segregation, and help manage proteotoxic stress. The identified proteolysis-related proteins were also (a) overexpressed in AR-low TNBC (Supplementary Figure S1) and AR-low BC (Supplementary Figure S3), and (b) showed a pattern of expression positively associated with high proliferation in TNBC (Figure 1) and BC more broadly (Supplementary Figure S5). All five proteolysis-related proteins are established transcriptional targets of the MMB-DREAM complex. Three out of these five ubiquitin–proteasome pathway genes are also ranked among the highest in the UALCAN TCGA database ranking of genes most overexpressed in TNBC, with UBE2C ranked 7th most overexpressed, UBE2T ranked 65th most overexpressed, and UBE2S ranked 106th most overexpressed in all TNBCs. Again, these elevated rankings suggest that their overexpression may contribute significantly to the pathobiology of AR-low TNBCs.

Ubiquitination is a post-translational modification of proteins that involves the addition of an evolutionarily conserved small protein, ubiquitin (Ub) or ubiquitin-like proteins, to lysine side-chains of target (abnormal or inherently short-lived) proteins destined for degradation by the proteasome [216,217]. In addition to marking proteins for degradation, ubiquitination can have different biological outcomes such as altering target proteins’ localization and/or their activity, and promoting or interfering with protein interactions [218,219,220]. As a result, dysregulation of this process can profoundly impact protein stability, activity, and localization. By regulating the persistence and stability of oncogenic or tumor suppressor proteins, the ubiquitination pathway can have tumor-suppressing or tumor-promoting effects. Ubiquitin-dependent protein degradation involves the E1, E2, and E3 enzymes: The C- terminus of Ub is first activated by an E1 (ubiquitin-activating) enzyme and is then transferred onto the active site of an E2 Ub-conjugating enzyme. Subsequently, E3 Ub ligases mediate the transfer of Ub from the active site of the E2 onto a specific lysine residue in the target protein [221]. E2s control crucial aspects of this cascade. Ubiquitin-conjugating enzymes E2S (UBE2S), E2C (UBE2C), and E2T (UBE2T) are important members of the E2 family and have been implicated in the tumorigenesis and progression of many cancers. PSMD14 is a deubiquitinase enzyme, while TUBA1B is a core player in aggrephagy—a process that removes harmful protein aggregates that arise due to mutations, incomplete mRNA translation, post-translational misfolding, improper protein modifications, and oxidative stress—which impacts tumorigenesis and cancer progression. To elucidate how the overexpression of these genes may contribute to the aggressive clinical phenotype of AR-low TNBCs, we conducted a detailed analysis of their established roles in proteolysis, as outlined in Supplementary Table S3 (which includes citations [222,223,224,225,226,227,228,229,230,231,232,233,234,235,236,237,238,239,240,241,242,243,244,245,246,247,248,249,250,251,252,253,254,255,256,257,258,259,260,261,262,263,264,265,266,267,268,269,270,271,272,273,274,275,276,277,278,279,280,281,282,283,284,285,286,287,288,289]). Taken together, overexpression (due to mutations or copy number alterations) of these Ub-proteasome- and aggrephagy-related genes may result in dysregulation of substrate degradation kinetics and proper mitotic progression, undermine normal mitotic checkpoints and safeguards, compromise DNA repair fidelity, and disrupt the balance between protein degradation and stabilization. This convergence contributes to the etiology and progression of cancer and promotes ongoing CIN, which fuels tumor evolution, intra-tumoral heterogeneity, and therapy resistance. Three out of the five protein degradation-related genes we focused on were found in the list of top 100 most overexpressed genes among highly functional aneuploid breast tumors in TCGA [76]: UBE2C ranked 6th, UBE2T was ranked 46th, and UBE2S was ranked 47th in this list. The CIN75 gene signature includes UBE2C [73]. Thus, AR-low TNBCs that overexpress these genes are likely to exhibit high levels of aneuploidy and CIN, which is a fuel for aggressive tumor phenotypes.

Our findings and data in the literature support the following model of the dysregulation that undergirds this FOXM1-mediated highly proliferative phenotype of AR-low TNBC. Normally, the expression of G1/S and G2/M cell cycle genes is tightly controlled by dynamic transcriptional complexes, ensuring orderly DNA replication and mitotic progression. However, in AR-low TNBC and TP53-mutant breast tumors, this tight regulatory balance is disrupted, and induces a transcriptional program that normally regulates proliferation to be pushed into overdrive. Central to this dysregulation is upregulation of a collaborative axis formed by (a) the transcription factor FoxM1, (b) WDR5—a core subunit of histone methyltransferase complexes that catalyze H3K4me3, and (c) the mitotic protein ASPM. When overexpressed, these three proteins drive the persistent upregulation of a set of genes, including 15 “core cell cycle-related genes” essential for proliferation. WDR5 epigenetically modifies the chromatin landscape of the FoxM1 promoter to license persistent FoxM1 transcription, enabling tumor cells to accumulate high levels of this oncogenic transcription factor. Our analyses showed that the expression of WDR5 shows a statistically significant negative correlation with the expression of AR (r = −0.40, *p* < 0.001), and a statistically significant positive correlation with the expression of FoxM1 (r = 0.60, *p* < 0.001), among TNBCs. While high levels of WDR5 transcriptionally ensure FoxM1 abundance, ASPM stabilizes FoxM1 protein and amplifies FoxM1 oncogenic activity through (i) phase separation into nuclear condensates that sustain persistent G2/M transcriptional activity, and (ii) feedback regulation. Furthermore, FoxM1 transcriptionally activates ASPM, creating a positive feedback loop in which each protein sustains the other. Consistent with this, we found that the expression of ASPM shows a statistically significant negative correlation with the expression of AR (r = −0.50, *p* < 0.001), and a statistically significant positive correlation with the expression of FoxM1 (r = 0.81, *p* < 0.001), among TNBCs. Once upregulated, FoxM1 binds to the MMB-MuvB complex and is activated through phosphorylation by CDK1 and PLK1. This active MMB–FoxM1 complex drives robust transcription of G2/M genes, including kinesins (KIF11, KIF14, KIF4A, KIF2C, KIF20A), centromere-associated proteins (CENPA, CENPO, CENPL, CENPF, OIP5), and cell cycle proteolysis regulators (UBE2C, UBE2S, UBE2T, PSMD14). In healthy cells, the precise regulation of these highly consequential target genes ensures accurate chromosome partitioning and genomic stability. In tumors, however, the overexpression of these effectors can promote unchecked proliferation accompanied by erroneous chromosome segregation, and fuel CIN and aneuploidy—processes strongly linked to tumor evolution, therapy resistance, and poor patient survival. ASPM itself regulates spindle orientation and microtubule minus-end dynamics, and its aberrantly elevated expression further compounds the chromosome mis-segregation driven by FoxM1 target gene overexpression. In this context, (i) loss of AR-mediated FoxM1 repression (via SPDEF), and (ii) disruption of the p53–p21–DREAM axis that normally restrains FoxM1 expression, further spike FoxM1 transcription, and increase production of its target effectors. Normally, p53 serves as the “guardian of ploidy”, eliminating aneuploid cells through apoptosis or senescence. In TP53-deficient tumors, however, cells with segregation defects survive and propagate. Collective upregulation of the 15 MMB-FoxM1 target genes we studied (and synergy with p53 deficiency, if it exists) locks cells into a high-proliferation state, with extensive CIN and aneuploidy, and engenders heightened transcriptional chaos and intra-tumoral heterogeneity that underlie the aggressive tumor biology of AR-low TNBC and TP53-mutant BCs. We rationally designed a weighted gene expression signature comprising the 15-gene set we studied, FoxM1, WDR5, and ASPM, and found that high levels of expression of this 18-gene signature was associated with poor prognosis in both BCs in general (Supplementary Figure S6, left panel) as well as specifically in TNBCs (Supplementary Figure S6, right panel), lending credence to the idea that this signaling network may lead to poor outcomes when in overdrive.

Our study primarily addresses a transcriptional mechanism wherein increased FOXM1 activity is produced by the synergistic actions of chromatin and nuclear condensate-regulatory factors, and factors that enhance FoxM1’s half-life (WDR5 and ASPM). To enrich our understanding of the mechanisms underlying FoxM1 upregulation, we also assessed the frequency of copy-number alterations (specifically, of gene amplification) among invasive breast carcinomas using datasets from cBioPortal (https://www.cbioportal.org/, last accessed on 6 February 2026) and found that FOXM1 exhibits amplification in a subset of breast tumors (Supplementary Table S4). Moreover, WDR5, ASPM, and all the genes in our cell cycle-related 15-gene set also show varying levels of gene amplification among breast tumors (Supplementary Table S4). These data indicate that copy-number gain is a plausible contributor to elevated FOXM1 pathway output in at least a fraction of cases. While our study focused on the transcriptional upregulation of FoxM1 by the combined actions of WDR5 and ASPM, we cannot rule out that FoxM1 gene amplification could co-occur with WDR5-ASPM-mediated transcriptional upregulation, and these two mechanisms need not be mutually exclusive. In fact, the same can be said of the 15 genes that are overexpressed downstream of FoxM1; their G2/M transcriptional upregulation by FoxM1-MMB complex could occur alongside the amplification of these genes. Thus, FoxM1 pathway hyperactivation in TNBC likely reflects both genomic (copy-number) and transcriptional/chromatin-based contributions, varying across tumors.

BCs with high proliferation and low AR-related signaling have a poor prognosis and unique molecular features with implications for therapy. This study aimed to build a granular portrait of AR-low TNBCs by diving deep into and contextualizing the patterns of dysregulation that drive and support their state of high proliferation. By enhancing our understanding of the processes and drivers undergirding the biology of these tumors, we hoped to unlock insights that will help better manage them. We uncovered that functional modules involving mitotic kinesin motors, centromere and kinetochore components, and proteolysis regulators may have an outsized impact on tumor biology and disease progression in AR-low TNBC and p53-deficient BC, because these emergent modules support and drive a highly proliferative state that is accompanied by mitotic errors and CIN.

CIN—the increased rate of whole or fractional chromosome gains or losses—may at first glance appear to be a feature that should adversely impact tumor cells’ fitness and impair their proliferation. However, CIN facilitates accelerated genomic evolution by generating diverse DNA copy number variants (CNVs) that can be selected during disease progression. The intercellular chromosomal heterogeneity that CIN produces proffers cancer cells the ability to generate and sample a variety of karyotypes in the cells’ quest to adapt to multiple stressors in their environment, while continually selecting for CIN-tolerance and a range of genotypes that may confer other advantages, including survival of aneuploid cells and their rapid proliferation. Several well-established prognostic signatures (e.g., Oncotype Dx, MammaPrint, PAM50, Meta-PCNA, GGI, CIN70, and the 12-gene genomic instability index) include genes that function in mitosis and are involved in cell cycle progression and are strongly associated with proliferation. Interestingly, they are also strongly associated with CIN and resistance to therapies. In a study that explored the complex relationship between CIN and proliferation, the authors developed a SNP array-based surrogate score used to assess the CIN status of a tumor, called the weighted genomic integrity index (wGII) [221]. This metric measures the percentage of gained and lost genomic material relative to the sample’s ploidy, avoiding bias from differing chromosome sizes. The authors found a significant positive correlation between increased chromosomal complexity (assessed by wGII) and the expression of these prognostic signatures. They also found that high CIN status is strongly correlated with increased expression of proliferation markers like MKI67 and the meta-PCNA gene set. The study identified “30 core regulators” whose expression and copy number are associated with increasing wGII scores; importantly, “core regulators” like UBE2C, UBE2T, and KIF14—also highlighted in this study—were amplified in high-CIN tumors and drove co-expression of proliferation modules. CIN genomes thus appear to select for amplifications in regulators such as UBE2C, establishing a “permissive landscape” for ongoing proliferation [221]. Understanding AR-low TNBCs as both high-CIN and high-proliferation (a) illuminates how intra-tumoral heterogeneity is generated in AR-low TNBC, and impacts key cancer phenotypes, and (b) highlights the importance of pursuing CIN attenuation by targeting its core regulators as a therapeutic strategy.

Our findings revealed how AR-low TNBC bends the machinations of the cell cycle at multiple levels and scales to drive, support, and propagate a state typified by more tumor cells entering and traversing the cell cycle, albeit in error-prone ways. Indeed, it appears that the benefits of the resulting intra-tumoral karyotypic and phenotypic heterogeneity outweigh the risks that accompany their aggressive proliferation and their faulty mitotic processes. The impacts of this dysregulation permeate so many aspects of the disease’s course, and this study represents a step forward in tallying them. Understanding what gene expression changes are required to adequately support high rates of proliferation, and what causes underlie the collateral errors and imperfections in the execution of cell division in these tumors, will pave the way for more stringent risk management of diverse cancers predisposed to greater intra-tumoral heterogeneity. While our finding that mitotic kinesin motors, centromeric proteins, and regulators of proteolysis are upregulated in AR-low TNBC was somewhat unsurprising, it also helped us unpack what a tumor needs in order to become highly proliferative. Our findings show how this tumor type manipulates cell cycle machinery, creating a state in which aggressive proliferation and genomic instability are not liabilities but assets for tumor evolution.

By highlighting “Cell cycle patterns” of gene expression enrichment and how dysregulation of each one touches and evokes dysregulation in others, this work strengthened our understanding of how phenotypes such as higher proliferation, CIN, chemoresistance, and disease progression then co-arise. This study has thus identified a network of vulnerability with therapeutic potential. Although FoxM1 is a well-established and promising anti-cancer target, no approved FoxM1-targeting drugs are currently available. However, small molecule inhibitors of FOXM1, such as NB73 and STL001, have shown promise in reducing tumor burden in preclinical studies, specifically in breast cancer [290,291,292]. Further preclinical investigation of these novel FOXM1 inhibitors in TNBC models, particularly of AR-low and TP53 mutant status, may support future clinical testing. An alternative FOXM1-signaling axis targeted approach includes disrupting FOXM1’s phase-separated state. Further investigation into this approach and other alternative FOXM1 signaling-targeted approaches could increase the success and translation of FOXM1-targeted therapies into clinical trials. Also, unlike the MMB-FOXM1 complex itself, mitotic kinesins possess enzymatic activity, making them “druggable targets.” This could lead to the development of more specific anti-mitotic drugs with potentially fewer side effects than traditional tubulin-targeting chemotherapies. Kinesin inhibitors, specifically targeting Eg5 (KSP) and KIF18A, are currently in phase I clinical trials for advanced solid tumors, with a focus on efficacy and safety as these targets are critical for normal cell division [293,294].

Our study is limited by separate analysis of AR-low and TP53-mutant TNBC tumors, which can occur as mutually exclusive events. Preclinical and bioinformatic validation of this FOXM1-regulated gene signature in TNBC tumors that are both AR-low and TP53 mutant would provide more accurate insight into the therapeutic potential of targeting this signaling axis in this particular tumor type. This study is also limited with lack of functional validation in preclinical models. Future preclinical studies validating upregulation and targeting of this FOXM1-mediated signaling axis in AR-low TNBC models will be critical to support future therapeutic intervention. While we acknowledge that transcriptional chaos and variations in levels of overexpression of these gene groups have a non-linear relationship with phenotypic outcomes, we also emphasize that the covariance of these specific cell cycle proteins tips the cell towards a certain state of increased proliferation. By (i) identifying coordinated dysregulation of mitotic motors, centromeric proteins, and proteolysis regulators as a defining signature of AR-low TNBC, (ii) connecting these modules mechanistically to CIN and aneuploidy, (iii) revealing a FoxM1–WDR5–ASPM axis that offers new conceptual and therapeutic entry points, insights from this study open avenues for developing targeted interventions aimed at dismantling the very circuits that sustain their malignant proliferation.

## 4. Materials and Methods

### 4.1. Gene Expression Correlation Analysis

The Targeted Correlation Analysis tool of bc-GenExMiner v5.2 [295] was used to assess pairwise gene expression correlations across publicly available BC transcriptomic datasets. This tool integrates annotated RNA-sequencing data from thousands of breast tumor samples and allows for the computation of Pearson’s correlation coefficients between the expression of a gene of interest and selected target genes within defined patient subgroups. Pearson’s pairwise correlation coefficients (r) and associated *p*-values were generated automatically by the Targeted Correlation Analysis module. Results were visualized as scatterplots and heatmaps using the built-in graphical interface to identify co-expression relationships.

### 4.2. Comparison of 15 Gene-Set Expression Between AR-Low vs. -High TNBC Tumors

QIAGEN OmicSoft Software V 12.9 was used to analyze differences in expression of 15 genes, regulated by FOXM1, between AR-low and AR-high tumors in two independent databases, METABRIC and Oncohuman. Only primary and metastatic tumor samples were selected. TNBC tumors were stratified according to a 50% cut-point for AR gene expression to sub-categorize into AR-low and AR-high subgroups. ANOVA F-test was used to analyze for significant differences in each gene.

### 4.3. Ranking of Overexpressed Genes in TNBC Tumors

The University of Alabama at Birmingham Cancer Data Analysis Portal (UALCAN) was used to determine the overexpression rankings of the genes of interest. UALCAN compares gene expression (TCGA level 3 RNA-sequencing) data between TNBC and other BC types, identifying and ranking the genes most significantly overexpressed in the TNBC subtype [109]. Genes with statistically significantly higher expression in TNBC samples are ranked based on the extent of their upregulation (fold-change).

### 4.4. Gene Expression Analysis Among Breast Tumors Based on Patients’ Self-Identified Race and TP53 Mutation Status

Gene expression analyses comparing normal breast tissue versus breast tumor samples, and analyses of breast tumors stratified by patients’ self-identified race and TP53 mutation status were conducted using the UALCAN, which accesses The Cancer Genome Atlas (TCGA) breast invasive carcinoma (BRCA) dataset. For each gene of interest, samples were grouped by self-identified race (e.g., African American, Asian, or Caucasian) and by TP53 mutation status (mutant vs. wild-type), and box-plot comparisons of log_2_-transformed transcript per million values were generated. Statistical significance of differential expression across sample subgroups was evaluated via UALCAN’s built-in Student’s *t*-test functionality (considering unequal variance).

### 4.5. Comparative Analysis of Promoter Methylation Levels

The UALCAN platform, which integrates Level 3 TCGA data, was used to analyze and compare promoter methylation levels for our genes of interest between normal breast tissues and breast carcinoma samples. The “TCGA Analysis” module of UALCAN accesses β-values derived from Illumina HumanMethylation450 BeadChip data, representing the proportion of methylated cytosines at CpG sites within gene promoters. For each gene of interest, methylation profiles (beta values) were visualized as box plots contrasting normal and primary tumor samples. Statistical significance of any observed differences was estimated using Student’s *t*-test, considering unequal variance.

### 4.6. Survival Analysis and Derivation of a Weighted Gene Signature

To evaluate the prognostic significance of individual genes, we performed univariate Cox proportional hazards regression analyses of publicly available microarray data using the Kaplan−Meier Plotter (KM Plotter) online tool [68] to identify the subset of “TNBC core genes” whose overexpression is associated with poorer RFS. The KM Plotter tool integrates gene expression and survival data from TCGA, Gene Expression Omnibus (GEO), and European Genome–Phenome Archive (EGA) datasets, enabling meta-analysis of transcriptomic profiles in relation to clinical outcomes. For each gene, patients were stratified into high- and low-expression cohorts based on the optimal cutoff determined automatically by the platform. All possible cutoff values within the interquartile range were assessed for each gene, and the cut-point that resulted in the lowest log-rank *p*-value was designated as the optimal choice by the platform. Hazard ratios (HRs) with 95% confidence intervals (CIs) and log-rank *p*-values were calculated to assess the association between gene expression and RFS. Kaplan–Meier survival curves were generated for visualization, and only results with log-rank *p* < 0.05 were considered statistically significant.

We assessed the prognostic value of a multi-gene expression signature using the Kaplan–Meier Plotter web tool. For each tumor cohort (all BCs or TNBCs only), we generated a weighted gene signature by combining the mean gene expression values of the selected genes (RNA sequencing data) into a single score using KM Plotter’s multigene weighted signature option (weights used: weight of 500 for WDR5, and weights of 20 used for KIF2C, CENPA, UBE2C, and UBE2S). Patients were stratified into high- versus low-signature groups using the tool’s cut-off optimization that minimized the log-rank *p*-value, and overall survival was evaluated by Kaplan–Meier analysis with hazard ratios and log-rank *p* values reported.

### 4.7. Analyses of Gene Expression Changes in TP53 Mutation Backgrounds Among Breast Tumors

The muTarget platform [68] was used to assess the impact of TP53 mutations on the expression of genes of interest in BC. This analysis utilized RNA-seq data and mutation calls from a TCGA cohort, comprising 305 TP53-mutant and 674 TP53 wild-type breast tumor samples. Genes were analyzed for differential expression between TP53 mutant and wild-type groups, restricted to mutations with a prevalence of ≥2% within the cohort to ensure statistical robustness. Statistical comparisons were performed using Welch’s *t*-test.

### 4.8. Tumor Microenvironment Analysis

To characterize immune infiltration patterns associated with overexpression of our genes of interest, we utilized the TIMER 3.0 web platform (http://timer.cistrome.org, last accessed on 13 July 2025), which allows comprehensive analyses of tumor–immune interactions using TCGA RNA-sequencing data of BC samples. Gene expression values were log_2_-transformed, and immune infiltration scores were computed using multiple deconvolution algorithms implemented in TIMER 3.0, including TIDE, XCELL, and CIBERSORT, to ensure robustness across analytical frameworks. Spearman’s correlation coefficients and corresponding *p*-values were calculated to assess the strength and significance of associations between gene expression and immune cell infiltration patterns. Data were visualized via heatmaps, allowing detailed evaluation of immune microenvironmental patterns in tumors with high expression of the target genes. We used a generative AI tool (ChatGPT 5.2, OpenAI) to assist with heatmap generation by inputting an Excel spreadsheet with Pearson’s pairwise correlation values and prompting the AI tool to generate a formatted heatmap (including standardized color scaling with “non-significant” values treated as zero and displayed as white, and annotated cell values/labels). All AI-generated outputs were reviewed and verified against the source spreadsheet.

## Data Availability

The original contributions presented in this study are included in the article/Supplementary Material. Further inquiries can be directed to the corresponding author.

## Supplementary Materials

The following supporting information can be downloaded at: https://www.mdpi.com/article/10.3390/ijms27041823/s1. References [296,297,298,299,300,301,302,303] are cited in the Supplementary Materials.
