# Supplementary material for: FOXM1 Signaling Network Transcriptionally Upregulates Expression of Proteins Involved in Mitotic Progression to Induce High Proliferation and Chromosomal Instability in Androgen Receptor-Low Triple-Negative Breast Cancer
Source: Int J Mol Sci. 2026 Feb 14;27(4):1823. doi: 10.3390/ijms27041823 (PMC12940972; doi:10.3390/ijms27041823)
Supplement: Supplementary file 1 [file ijms-27-01823-s001.zip › PROOFS—Suppl Tables—renumbered refs.pdf]

Suppl. Table S1: Overexpression of FoxM1-regulated mitotic kinesins promotes aggressive phenotypes.

Gene Name	Protein functions and phenotypes associated with under/overexpression
KIF14	• Kinesin family member 14 (KIF14) is a minus-end-directed motor protein that is localized to the cytoplasm during interphase, to the spindle poles and spindle microtubules during mitosis, and to the midbody during cytokinesis; it regulates key steps in spindle assembly, chromosome segregation and maintenance of genomic integrity, and cytokinesis [111].<br>• When KIF14 expression is suppressed, midbody cleavage fails to occur, inducing cytokinesis failure, and causes a delay in the metaphase-to-anaphase transition, characterized by misaligned chromosomes that oscillate abnormally between the spindle pole body and the metaphase plate, which suggests that KIF14 is essential for these processes [112].<br>• Aberrant KIF14 overexpression, often via gene amplification or transcriptional upregulation, has been reported in several malignancies, including breast, ovarian, and lung cancers, and is associated with poor clinical outcomes [113,114].<br>• In cancer cells, elevated KIF14 enhances proliferation, inhibits apoptosis, and promotes chromosomal instability, underscoring its role as an oncogenic driver and a potential therapeutic target [115].<br>• In BC, KIF14 overexpression was strongly associated with ER-negativity, and predicted worse outcomes [113,116].<br>• Specifically, in TNBC, high levels of KIF14 expression are associated with resistance to chemotherapy [104,117].<br>• Localization of KIF14 to the tips of distinct architectural arrangements of cells within breast tumors, known as "torpedo-like structures", was significantly associated with increased distant metastases and decreased metastasis-free survival in BC patients. The transcriptomic profiles of the KIF14-positive cells in these structures suggested a specialization for invasiveness; these KIF14-positive cells are likely metastasis initiating cells [118].
KIF11	• Kinesin family member 11 (KIF11/Eg5/Ksp) is a plus end-directed kinesin [119] involved in construction and maintenance of the bipolar spindle because of its role in crosslinking and sliding antiparallel microtubules [120,121].<br>• KIF11 is also the main force generator in centrosome separation, which is essential for bipolar spindle formation [119].<br>• KIF11 overexpression has been implicated in various cancers (gastric, bladder, renal cell, astrocytic, laryngeal squamous cell, oral cancer) and is often associated with advanced stage or poor prognosis [122].<br>• KIF11 has been identified as a potential oncogene that drives the development and progression of BC, strongly associated with poor patient

	outcomes [122,123]. Experimental inhibition of KIF11 significantly suppressed BC cell proliferation, migration, and invasion, while promoting apoptosis, both in vitro and in vivo [122].<br>• In BC, KIF11 is regulated by the Id proteins (specifically Id1 and Id3), that play key roles in sustaining cancer stem cell phenotypes [124]. KIF11 enhances the self-renewal of BC cells by activating the Wnt/β-catenin signaling pathway [125].<br>• Clinically, high KIF11 expression in TNBC is associated with shorter DFS [126]. KIF11 levels were markedly elevated in the CD44+/CD24− subpopulation of docetaxel-resistant TNBC cells. KIF11 knockdown reduced this cancer stem cell–like fraction, impaired mammosphere formation, and suppressed proliferation by inducing G2/M arrest followed by apoptosis. In docetaxel-resistant TNBC xenografts, a KIF11 inhibitor significantly limited tumor growth. Therefore, KIF11 is an established therapeutic target in chemo-resistant TNBC [126].
KIF4A	• Kinesin family member 4A (KIF4A), a plus-end-directed chromokinesin motor protein, binds both chromatin and microtubules, and plays an important role in chromosome condensation and segregation, central spindle formation, spindle midzone organization, and cytokinesis [112127–129].<br>• KIF4A overexpression has been implicated in driving a poor prognosis in several cancer types including lung cancer, cervical cancer, hepatocellular carcinoma, and oral cancer [106].<br>• In BC specifically, KIF4A overexpression has been extensively documented to predict a poor prognosis [130–133].<br>• KIF4A rapidly accumulates at sites of DNA damage where it binds to BRCA2 and modulates the BRCA2/Rad51 DNA damage response pathway while inhibiting the enzymatic activity of PARP-1; the result is high levels of genomic instability in KIF4A-overexpressing tumors, and disease progression [134].<br>• A study that aimed to identify a circRNA-miRNA-mRNA competing endogenous RNA (ceRNA) regulatory network involved in EMT in BC cells, identified two circRNAs (hsa_circRNA_002082 and hsa_circRNA_400031), which act as ceRNAs, and sponge up 10 specific miRNAs and, consequently, prevent these miRNAs from regulating their 6 target mRNAs, including KIF4A, CENPF, and OIP5 mRNAs. The study identified KIF4A, CENPF, and OIP5 as among the "hub gene" targets that are significantly overexpressed in BC and are implicated in disease progression and worse patient prognosis [135].<br>• CircKIF4A interacts with EIF4A3 to stabilize SDC1 mRNA, which activates the c-src/FAK signaling pathways and promotes TNBC progression; thus, KIF4A upregulation plays a critical role in TNBC progression [136].
KIF2C	• The kinesin-13 family member 2C (KIF2C) is a plus end-kinesin motor and is also known as the mitotic centromere-associated kinesin (MCAK) [150].

	KIF2C is localized on the centromere, and KIF2C activity maintains genomic stability by ensuring proper kinetochore-microtubule attachments [108].<br>• KIF2C regulates microtubule dynamics, especially during mitosis, and uniquely, depolymerizes microtubules by disassembling tubulin subunits at the polymer ends; as a result, KIF2C can mediate ciliary disassembly by depolymerizing microtubules within cilia [137].<br>• KIF2C depletion or down-regulation led to prominent defects in chromosome congression and segregation due to improper kinetochore attachments in potoroo kidney cells [138].<br>• Conversely, KIF2C overexpression promoted microtubule depolymerization, resulting in increased microtubule detachment from centromeres [139].<br>• Fine-tuned regulation of KIF2C is important for cell motility and migration due to its effects on the actin-microtubule interactions and cytoskeletal dynamics, and turnover of focal adhesions. Deregulation of KIF2C impairs cell motility and leads to severe mitotic defects and chromosomal instability [140].<br>• Kif2C knockdown or knockout led to accumulation of endogenous DNA damage, DNA damage hypersensitivity, and reduced double stranded break repair via both non-homologous end-joining and homologous recombination; KIF2C thus plays an important role in DNA damage repair and maintenance of genomic integrity [141].<br>• Among BCs, KIF2C was upregulated across all molecular subtypes. High KIF2C expression was associated with poor OS in BC, across multiple datasets [107]. Moreover, high-KIF2C breast tumors showed high tumor mutational burden and an immune cell infiltration profile that predicted a more favorable response to immunotherapy. Thus, KIF2C is a potential prognostic biomarker and predictor of immunotherapy response in BC.<br>• High levels of KIF2C reduced doxorubicin sensitivity partly via enhanced autophagy. Overexpressed KIF2C also associated with pyruvate kinase M2 (PKM2) and curtailed the latter's ubiquitination, enhancing PKM2 stability and reinforcing glycolysis/the Warburg effect that undergirds the chemo-resistant phenotype [142].<br>• Thus, KIF2C upregulation drives doxorubicin resistance, proliferation, migration, and invasion in BC by stabilizing PKM2 and promoting glycolysis [142].
KIF20A	• KIF20A (also known as MKlp2 and RAB6-KIFL), a member of the kinesin superfamily-6, is a plus-end-directed motor protein that localizes to the cleavage furrow, intercellular bridge, and midbody. During telophase/cytokinesis, KIF20A shows strong localization to the cell cortex at the equator and the midbody, and this localization is essential for the proper formation of the cleavage furrow and execution of cytokinesis [143].<br>• KIF20A relies on myosin-II for its localization to the equatorial cortex, which is in turn required to (i) recruit Aurora B to the equatorial cortex, (ii) promote

	the highly focused accumulation of active RhoA at the equatorial cortex and stable ingression of the cleavage furrow during cytokinesis. KIF20A-mediated targeting of Aurora B to the cell cortex at the equator, and the formation of a complex between KIF20A and actomyosin filaments, is essential for the maintenance and progression of the ingressing furrow, and successful completion of cytokinesis [144].<br>• KIF14, KIF4A, and KIF20A collaborate in the formation and function of the central spindle [111,145].<br>• Plk1 also phosphorylates KIF20A, which increases KIF20A's affinity for microtubules, facilitating its attachment and function during late mitosis [146].<br>• In the context of BC, high KIF20A expression showed a strong correlation with more aggressive features, such as positive lymph nodes, larger tumor size, high histological grade and Ki67 labeling index; high KIF20A expression was in fact, an independent predictor of poor OS [107,147].<br>• Inhibition of KIF20A led to marked reduction in proliferation and invasion of BC cells [107].<br>• KIF20A overexpression is associated with therapy resistance in BC. Increased post-radiotherapy levels of KIF20A were linked to higher recurrence rates in BC [147].<br>• Among BC patients who received anthracycline and/or taxane-containing neoadjuvant chemotherapy, a decreased pCR rate was observed in patients with high KIF20A expression [147].<br>• Genes associated with multi-drug resistance in cancer treatment (ABCB1, ABCC1, ABCG2) were co-overexpressed with KIF20A [147].<br>• Upregulation of KIF20A also plays a role in doxorubicin resistance in BC cells [148].<br>• In TNBC cells, KIF20A knockdown significantly reduced cell viability, proliferation, migration, and invasion [149].

Suppl. Table S2: Overexpression of FoxM1-regulated centromeric proteins promotes aggressive phenotypes.

Gene Name	Protein functions and phenotypes associated with under/overexpression
CENPA	• Centromeres are defined epigenetically by the presence of a centromere-specific histone H3 variant CENPA. CENP-A plays an indispensable role in centromeric chromatin assembly and centromere specification, in addition to being essential for kinetochore assembly and proper chromosome segregation [150,151].<br>• CENPA is deposited exclusively in G1 of the cell cycle by a dedicated histone chaperone HJURP (Holliday junction recognition protein) to replace its

	canonical counterpart, and forms specific CENPA nucleosomes with histone H4, H2A, and H2B; CENPA-containing nucleosomes are interspersed with canonical histone H3-containing nucleosomes in centromeres. [152–157].<br>• Kinetochores regulate chromosome movements during mitosis, act as a central platform for signaling factors that govern the fidelity of chromosome segregation, and are composed of two large submodules: the inner and outer kinetochore [158]. The inner kinetochore, assembles on centromeric chromatin, serves as a structural platform for outer kinetochore assembly, and persists with centromeres throughout the cell cycle [157]. The outer kinetochore assembles only during mitosis and plays an essential role in generating and sensing microtubule attachments [159]. The outer kinetochore is quickly dismantled once mitosis concludes. Kinetochore dysfunctions commonly lead to CIN and aneuploidy [160,161]. CENPA recruits inner kinetochore proteins, that ultimately connect to outer kinetochore proteins, that in turn, interact with spindle microtubules to drive accurate chromosome segregation [162].<br>• Although the centromeric environment generally suppresses transcriptional initiation, active RNA polymerase II complexes, centromere-derived RNAs (cenRNAs), and nascent transcripts have been reported to co-localize with centromeric chromatin [159]. cenRNAs are necessary for the proper formation of CENP-A-containing centromeres [163–166] and for the formation of pericentromeric heterochromatin [167,168]. The transcripts also play a role in the structure and function of the centromere–kinetochore interface [169]. CENPA is an m6A reader of cenRNA, and CENPA's m6A reading ability epigenetically governs centromere integrity [169].<br>• When the centromere is unable to form stable connections to the mitotic spindle, aneuploidy results. CENPA maintains the integrity of centromere-associated repetitive sequences by ensuring their effective replication in human cells [183]. CENPA-containing centromeric chromatin is specialized to facilitate DNA replication and maintain the integrity of transcribed, noncoding, repetitive centromeric DNA during S phase, and suppress chromosome translocations and their deleterious sequelae [170].<br>• Overexpressed CENPA mislocalizes to non-centromeric regions of chromosomes, causing CIN [171].<br>• The proper levels, localization, and function of CENPA ensures genome stability.
CENPO	• Centromere protein O (CENPO) is a structural centromere protein that plays vital roles in cell proliferation and is essential for several critical aspects of mitosis including centrosome separation and bipolar spindle assembly, kinetochore assembly, accurate chromosome segregation, and checkpoint signaling during mitosis [157,172–174].

	• CENPO is a component of the CENPA-CAD (nucleosome distal) complex (comprised of CENPI, CENPK, CENPL, CENPO, CENPP, CENPQ, CENPR and CENPS, which are all purified in association with CENPA nucleosomes). The CENPA-CAD complex interacts with the CENPA-NAC complex (that includes CENPA, CENPC, CENPH, CENPM, CENPN, CENPT and MLF1IP/CENPU). CENPA-CAD/NAC cooperatively modulate the kinetochore-bound levels of the NDC80 complex (that is essential for microtubule–kinetochore attachment and spindle checkpoint signaling), and they collaborate to drive efficient chromosome segregation during mitosis [173].<br>• CENPO is also involved in incorporation of newly synthesized CENPA into centromeric nucleosomes [175].<br>• CENPO overexpression in gastric cancer was associated with high clinical stage, tumor volume, lymph node metastasis, and shorter survival times [176]. CENPO overexpression in ovarian cancer cells drives abnormal proliferation and resistance to apoptosis [177]. Overexpression of CENPO in colorectal cancer was associated with high proliferation [178] while that in bladder cancer was associated with disease progression [179]. In lung adenocarcinoma, CENPO expression correlates with age and advanced TNM stage, and patients with high CENPO expression have poorer OS and DFS [180].<br>• Dysregulation of CENPO leads to CIN, a hallmark of cancer, driving aggressive tumor behavior in diverse malignancies [181].
CENPL	• As a component of the CENPA-CAD complex, CENPL is involved in the recruitment of CENPA and in the assembly of centromeric chromatin [157,175].<br>• CENPL, along with other members of the CENPA-CAD complex, also helps assemble kinetochores on top of CENPA-containing centromeric chromatin, to ensure proper chromosome segregation during mitosis. The CENPA-CENPL-CENPO complex interacts with the microtubule motor proteins during the transition from metaphase to anaphase, ensuring accurate alignment of chromosomes on the spindle and their proper segregation. Dysfunction of the CENPA-CENPL-CENPO complex leads to defective assembly of the kinetochore, misalignment of chromosomes, and eventually to CIN [155].<br>• CENPL is proposed to be an oncogene and BC is among several cancer types exhibiting elevated CENPL expression [155]. CENPL overexpression has been shown to drive chemoresistance in BC cells [156].<br>• High-CENPL breast tumors showed hyperactivation of several oncogenic pathways, especially those related to proliferation [157].

CENPF	• CENPF is a very large kinetochore protein that plays a critical role in the dynamic attachment of kinetochores to spindle microtubules, the proper segregation of chromosomes during mitosis, vesicular transport, and even in ciliopathies [158–163].<br>• CENPF gradually accumulates during the cell cycle until it reaches peak levels in G2 and M phase cells and is rapidly degraded upon completion of mitosis [164].<br>• CENPF interacts with downstream microtubule motors such as dynein, stabilizing the kinetochore-microtubule attachment necessary for chromosome segregation. It also coordinates in a complex manner with other kinetochore-associated proteins to ensure integrity of the spindle. Disruption of CENPF can lead to defects in chromosome segregation, leading to misalignment of chromosomes or aneuploidy [1322].<br>• In a cancer-specific context, co-occupation of the promoters of centromeric proteins by BMYB-FOXM1 was insufficient to initiate the full FOXM1-associated transcriptional program. CENPF as a crucial co-regulator with FOXM1 in orchestrating G2/M gene expression and ensuring accurate chromosome segregation. Notably, this FOXM1–CENPF collaboration appears to selectively co-regulate a subset of G2/M genes to promote cell proliferation [165].<br>• High-resolution electron tomography of the kinetochore–microtubule interface has revealed that the plus-ends of kinetochore-attached microtubules often exhibit flared protofilaments, formed by outwardly curved tubulin strands. These curled protofilaments appear to connect to centromeric chromatin via fine fibrillar linkages. Such structural features suggest the involvement of filamentous kinetochore proteins that preferentially bind to curved tubulin assemblies. These interactions likely play a critical role in mitotic force generation, as most chromosomes move toward the spindle poles during anaphase by maintaining strong attachments at the plus-ends of kinetochore microtubules. CENP-F binds more effectively to curved tubulin oligomers than to intact microtubule lattices, and both of its Microtubule-binding domains can engage with dynamic microtubules to harness the mechanical energy generated during tubulin depolymerization [166].<br>• CENPF mis-regulation has been linked to progression of several types of cancer including papillary thyroid cancer [167].<br>• CENPF is overexpressed in BC, where it promotes proliferation, migration, and invasion [168]. CENPF overexpression promotes BC bone metastasis by activating PI3K-AKT-mTORC1 signaling [169]. High CENPF expression portended worse RFS in BC patients receiving neoadjuvant chemotherapy [170]. CENPF was also one of the five embryonic stem cell-specific genes whose expression predicts a high risk of BC recurrence [171]. CENPF is part

	of a 14-gene genomic signature of metastatic relapse after adjuvant FEC100 regimen (5-fluorouracil 500 mg/m(2), epirubicin 100 mg/m(2) and cyclophosphamide 500 mg/m(2)) [172]. In BC, high CENP-F expression, correlated with higher standardized uptake value (SUV) detected by 18F-fluorodeoxyglucose positron emission tomography/computed tomography (FDG PET/CT); SUV correlates with proliferation of primary BC, and tumoral SUV levels may serve as a pretherapeutic indicator of aggressiveness of BC [173].<br>• In TNBC, CENPF is overexpressed and high CENPF expression was associated with chemotherapy resistance; conversely, silencing CENPF increased chemosensitivity [170]. CENPF regulates TNBC chemoresistance through the RB-E2F1-Chk1 axis.<br>• CENPF also promotes TNBC metastasis [174].<br>• CENPF silencing exacerbates arachidonic acid metabolism-induced ferroptosis in TNBC cells, suggesting that CENPF normally inhibits ferroptosis [174]. Furthermore, E2F1 binds to CENPF promoter and activates CENPF expression, which then leads to suppression of ferroptosis. Importantly, arachidonic acid metabolism-induced ferroptosis suppresses TNBC metastasis. The binding of PSMD14 (a deubiquitinase) to E2F1 stabilizes E2F1, which then results in upregulation of CENPF expression, suppression of ferroptosis, and increased metastasis in TNBC. Thus, overexpression of CENPF and PSMD14 can synergize to promote metastasis in TNBC [213]; this is extremely important given that PSMD14 is also one of the 15 FoxM1-regulated genes we focused on in our study.
OIP5	• OIP5 gene encodes a 25-kDa protein that was originally identified in a yeast two-hybrid screen for proteins that interact with Opa (Neisseria gonorrhoeae opacity-associated) proteins [175].<br>• OIP5's main function deals with the loading of CENP-A onto centromeric chromatin by collaborating with HJURP [176]; this function is important for the structure, stability, and function of the centromere throughout the cell cycle.<br>• The fidelity of chromosome segregation in mitosis depends on OIP5, because defects in the function of OIP5 may lead to aberrant alignment and attachment of kinetochores and precipitate chromosome missegregation and aneuploidy [177,178].<br>• OIP5 is historically well established as a Cancer/Testis (C/T) antigen that belongs to a unique class of tumor-associated antigens with expression normally restricted to immune-privileged sites [179,180].<br>• OIP5 is normally expressed in the testis and at low levels in bone marrow, thymus, and colon [181], but is markedly overexpressed in BC [177], glioblastoma [182], colorectal [183], bladder [184], gastric cancers [185], oral cancer [186], and lung and esophageal cancers [187].

	• The transient expression of OIP5 in NIH3T3 cells resulted in a 2-fold increase in proliferation rate, highlighting its oncogenic properties [183].<br>• In BC, OIP5 expression showed a significant positive correlation with advanced clinical stage [188], and its knockdown inhibited the proliferation of BC cells, promoting apoptosis.<br>• Mechanistically, OIP5 is a direct target of miR-139-5p, and upregulation of OIP5 mRNA turns it into an endogenous molecular sponge that mops up miR-139-5p. Notch1 is a target of regulation by miR-139-5p; as a result, OIP5 overexpression leads to overexpression of Notch1 [188].

Suppl. Table S3: Overexpression of FoxM1-regulated proteins that are involved in protein degradation pathways, promotes aggressive phenotypes.

UBE2C	• UBE2C is an E2 (Ub-conjugating) enzyme required for the degradation of mitotic regulators in cooperation with the anaphase-promoting complex/cyclosome (APC/ C) [222–225].<br>• UBE2C initiates the Ub chain on the target proteins via the K11 linkage.<br>• Two cell culture–based studies also revealed that UBE2C inhibits autophage [226,227].<br>• Transgenic mice overexpressing UBE2C were prone to developing carcinogen-induced lung tumors and a broad spectrum of spontaneous tumors [225].<br>• High expression of UBE2C is found in many human cancers of the brain, lung, cervix, colon, liver, thyroid, breast, and nasopharynx, and depletion of UBE2C from cancer cells significantly reduces proliferation and induces apoptosis [228,229].<br>• The overexpression of UBE2C and the association between high expression of UBE2C and poor prognosis in BC is well documented [230–235]. FOXM1, BMYB, and UBE2C are all significantly overexpressed in high-grade breast tumors [182]. In BC overall, UBE2S and UBE2C are overexpressed while Numb, the cell fate determinant and tumor suppressor, is downregulated [240]. Numb regulates other tumor suppressors, such as p53 and PTEN, and promotes GLI1 oncogene degradation via ubiquitination [236–239]. In BC, Numb acts as a tumor suppressor and is a negative regulator of EMT in both human mammary epithelial cells and BC cells [240]. Reduced NUMB expression was significantly associated with elevated EMT in TNBC [241]. In normal mammary epithelial cells and BC cells expressing wild-type p53, NUMB suppresses EMT by stabilizing p53. However, in TNBC cells, loss of NUMB promotes EMT through the activation of Notch signaling pathways. Supporting this mechanism, clinical data reveal a strong association between low NUMB levels, elevated Notch signaling, and the TNBC subtype [241]. Our analysis using the bc-GenexMiner platform also showed that among TNBCs, expression of Numb shows a statistically significant negative correlation with the expression of UBE2S ($r = -0.34$, $p<0.0001$, $n = 293$) and UBE2C ($r = -0.31$,

	p<0.0001, n = 293), and a statistically significant positive correlation with the expression of AR (r = 0.26, p<0.0001, n = 293), raising the possibility that AR-low TNBC exploits an analogous mechanism to induce EMT and facilitate metastasis.<br>• UBE2C facilitates malignant behavior in lung adenocarcinoma cells by ubiquitin-dependent degradation of p53 to suppress the p53/p21 signaling pathway [242].<br>• UBE2C is transcriptionally repressed by wild-type p53 [249]. Importantly, wild-type p53-mediated inhibition of UBE2C is p21-E2F4-dependent. DNA damage-induced wild-type p53 leads to spindle assembly checkpoint arrest by repressing UBE2C. UBE2C acts in G2/M checkpoint control, and plays a fundamental role in the maintenance of genetic stability by regulating the degradation of securin, a protein that impairs the premature segregation of chromosomes by binding to and inhibiting the enzyme separase [234]. Thus, UBE2C upregulation exacerbates the phenotypes induced by TP53 loss [243].<br>• CLDN19 as an upstream negative regulator of UBE2C in BC cells [244]. CLDN19 expression was significantly reduced in breast tumor tissues [244] and is associated with UBE2C overexpression and with poor patient survival. in BC cells with low CLDN19 expression, the extracellular matrix (ECM) is a 3D environment that can activate Wnt signaling. Under normal conditions, CLDN19 suppresses UBE2C, thereby preventing ECM-induced activation of the Wnt/β-catenin oncogenic pathway. Thus, CLDN19 inhibits UBE2C-mediated Wnt signaling activation in response to ECM cues in three-dimensional or in vivo contexts.<br>• Inhibition of UBE2C sensitizes BC cells to radiation, doxorubicin, and even hormone blocking agents [245].
UBE2S	• The human Ub-binding enzyme E2S (UBE2S), is associated with the APC/C and is essential for the Ub conjugation and elongation of Ub chains on target substrate proteins destined for degradation by the 26S proteasome. Once UBE2C attaches ubiquitin onto K11 in the target proteins, UBE2S promotes the elongation of ubiquitin chains thereby enabling substrate degradation to proceed [246].<br>• UBE2S executes pivotal functions during mitosis as it is responsible for the timely ubiquitination and degradation of several cell cycle regulators including mitotic cyclins [246,247].<br>• UBE2S becomes particularly important for efficient substrate degradation when APC/C activity has been compromised by prolonged spindle-assembly checkpoint (SAC) arrest. Following release from SAC arrest, UBE2S-depleted cells neither degrade crucial APC/C substrates, nor silence this checkpoint, whereas SAC bypass via BUBR1 depletion or Aurora-B inhibition negates the requirement for UBE2S [246].<br>• Cells that overexpress UBE2S degrade APC/C substrates more efficiently, facilitating mitotic exit even when it is not appropriate [246].

	• UBE2S also modulates the stability of the tumor suppressor VHL (Von Hippel-Lindau) under normoxic conditions by promoting VHL's ubiquitination and degradation [248,249]. VHL destruction then promotes metastasis and proliferation through the VHL/HIF-1α/STAT3 pathway [250].<br>• UBE2S is itself degraded through the proteasome pathway [251].<br>• UBE2S is overexpressed in several cancers and this overexpression is associated with cancer progression, resistance to chemotherapy, and poor prognosis. Aberrantly high expression of UBE2S was observed in cervical cancer, ovarian cancer (where high UBE2S upregulates Wnt/β-catenin signaling, leading to resistance to olaparib in vitro and in vivo, and enhanced proliferation triggered by upregulation of PI3K/AKT/mTOR signaling), BC, oral squamous cell carcinoma (where it promotes the degradation of p53 target, p21), endometrial cancer (where it downregulates Sox6 expression and promotes β-catenin signaling, cell migration, and proliferation), hepatocellular carcinoma or HCC (where it enhances the proteolysis of the tumor suppressor, p53), colorectal cancer (where it extends the half-life and promotes the accumulation of β-catenin), and kidney cancer [252–260]. UBE2S also participates in NHEJ-mediated DNA repair process, and thus modulates sensitivity to chemotherapy in glioblastoma [261]. In hepatocellular carcinoma cells, overexpression of UBE2S promoted proliferation, invasion, metastasis, and G1/S phase transition in vitro, and promoted tumor growth significantly in vivo. Mechanistically, UBE2S can enter the nucleus through its nuclear localization signal, where it interacts with TRIM28 (an E3 Ub-ligase); these two proteins work together to enhance the ubiquitination and degradation of the CDK inhibitor p27, thereby promoting cell cycle entry and progression [262]. Thus, UBE2S overexpression drives aggressive tumor biology through a variety of downstream pathways.<br>• UBE2S is considered to be a critical component of the pathway by which FoxM1 modulates resistance to cytotoxic agents in HCC cells, and potentially in other cancer types [263]. Overexpression of UBE2S is also associated with radiotherapy resistance because of UBE2S's involvement in DNA damage repair [251]. Inhibition of UBE2S enhances the susceptibility of cervical cancer HeLa cells to etoposide and adriamycin, and heightens the chemosensitivity to topotecan [251].<br>• In BC cells, UBE2S knockdown suppressed cell spreading, migration, and invasion [256]. UBE2S overexpression was also strongly associated with resistance to Topoisomerase II inhibitors in BC [252]. UBE2S is a component of the 4-gene Ub-related genes signature that was found to be an independent risk factor for poor overall survival among BC patients [264]. UBE2S was also one of the genes associated with improved response to neoadjuvant chemotherapy in BC [265]. In TNBC, UBE2S promotes proliferation and survival, particularly under conditions of prolonged SAC activation [266].

	• Together, these findings position UBE2S as a critical orchestrator of mitotic progression and tumor-promoting pathways, whose dysregulation may drive unchecked cell division, metastasis, and therapy resistance.
UBE2T	• The ubiquitin-conjugating enzyme E2T (UBE2T) plays a pivotal role in facilitating the repair of DNA damage, including within the Fanconi anemia pathway [267]. UBE2T monoubiquitinates several proteins of this pathway, including ANCD2 and FANCI [268].<br>• UBE2T also interacts with and colocalizes with the BRCA1/BRCA1-associated RING domain protein (BARD1) complex, and is involved in BRCA1 downregulation [269].<br>• UBE2T-driven ubiquitination influences multiple cancer-associated signaling pathways: ubiquitination of AKT by UBE2T activates the AKT/β-catenin signaling cascade, while ubiquitination of β-catenin promotes β-catenin's nuclear localization [270]. Furthermore, in lung adenocarcinoma, UBE2T enhances autophagy through modulation of the p53/AMP-activated protein kinase (AMPK)/mammalian target of rapamycin (mTOR) axis [271].<br>• UBE2T is overexpressed in BC, and its overexpression predicts poor prognosis. In BC cells, UBE2T mediates resistance to DNA replication stress, apoptosis, and chemotherapeutic agents, and inhibition of UBE2T augments the growth-inhibitory effects of agents that induce DNA replication stress [272]. A recent study that examined breast tumors with an extreme dependence on DNA repair machinery identified DNA repair networks that are in overdrive in BC. UBE2T was among five genes (a) that were found to be amplified across all BC subtypes, and (b) whose overexpression was consistently associated with unfavorable clinical outcomes regardless of molecular subtype [273]. Another study utilized a novel strategy for predicting risk of BC metastasis and identifying key genomic biomarkers through the integration of machine learning (ML) and explainable artificial intelligence (XAI), to enhance interpretability and address the "black box" limitation of complex ML systems. Elastic net feature selection identified 18 strong biomarker candidates for BC metastasis. Among these, elevated UBE2T expression correlated with an increased likelihood of metastatic progression [274].<br>• UBE2T was also identified as a key regulator of BC stem cell (BCSC) stemness. Genetic ablation of UBE2T markedly impaired BCSC stemness. UBE2T partners with the E3 ubiquitin ligase TRIM25 to catalyze polyubiquitination and degradation of CBX6. Reduced CBX6 levels facilitate transcriptional activation of SOX2 and NANOG (which are transcription factors included among the "core pluripotency network" that regulates stem cell self-renewal and differentiation), thereby enhancing BCSC stemness. These findings define the UBE2T–TRIM25–CBX6 signaling axis as a critical modulator of BCSC stemness [275].

	• Aberrantly high levels of UBE2T in BC also enhance tumor growth, proliferation, migration, invasion, and glycolysis through the PI3K/AKT signaling pathway [276].<br>• In TNBC, UBE2T overexpression promotes brain metastases; UBE2T upregulation is therefore associated with poor prognosis. Normally, a CDC42-mediated autophagy-dependent pathway promotes the trafficking of the immune checkpoint protein CD276 to lysosomes for degradation; by mediating the ubiquitination and proteasomal degradation of the Rho GTPase CDC42, UBE2T overexpression increases CD276 levels, impairs the antitumor activity of CD8+ T cells, and enables tumor immune escape. Inhibition of UBE2T raises the TNBC sensitivity to immune checkpoint blockade and suppresses BC brain metastases [277]. Higher UBE2T expression levels were predictive of a lower rate of pathological complete response in TNBC patients following neoadjuvant chemotherapy [278].
PSMD14	• The 26S proteasome non–ATP regulatory subunit 14 PSMD14 (also known as RPN11 and POH1) is a deubiquitinating enzyme that plays a significant role in homeostasis, and is implicated in differentiation, pluripotency, the DNA damage response, cellular proliferation, and senescence [2879<br>• PSMD14 overexpression is implicated in the pathology of various cancers, including liver, esophageal, and BC [280–283], where PSMD14 overexpression is associated with adverse clinical outcomes.<br>• Amplification of the PSMD14 gene can lead to increased expression levels. This amplification is often associated with CIN in cancer cells [284].<br>• PSMD14 knockdown caused cell arrest in the G0-G1 phase, and ultimately led to senescence in a broad range of cancer cell lines [285].<br>• PSMD14 is a key regulator of the stability of IRF3 (a critical transcription factor in antiviral innate immune signaling) and type I interferon (IFN) signaling. PSMD14 counteracts autophagic degradation of IRF3 by deubiquitinating IRF3, thereby preserving basal IRF3 levels and sustaining IFN activation [286].<br>• PSMD14 is one of seven genes that comprise a multivariable prognostic model in BC; these 7 genes were identified as being DNA damage response-related genes that were differentially expressed between high- and low-Tumor mutational burden (TMB) breast tumors in the TCGA dataset, and whose overexpression was associated with poor outcomes [287].<br>• In TNBC, PSMD14 is markedly upregulated and drives tumor cell proliferation, migration, and invasion [294]. In TNBC, PSMD14 deubiquitinates SF3B4 (an RNA-binding protein, or RBP, involved in pre-mRNA splicing), stabilizing the protein and enabling it to form a complex with heterogeneous nuclear ribonucleoprotein complex (HNRNPC), which acts both as an RBP, as well as an m^6A reader. The SF3B4-HNRNPC complex then binds m^6A-modified FADS1 mRNA, promoting exon 10 inclusion and upregulating FADS1 expression. Elevated FADS1, in turn, activates the Akt/mTOR pathway [288].

	• In TNBC, PSMD14 also suppresses arachidonic acid metabolism–induced ferroptosis through activation of the E2F1/CENPF signaling axis. PSMD14 acts as a central driver of TNBC progression through ferroptosis inhibition and metastatic pathway activation [174].
TUBA1B	• Tubulin α-1b chain (TUBA1B) is an important α-tubulin isoform; this protein is therefore a major component of the microtubule cytoskeleton and plays critical roles during chromosome segregation and mitotic progression. TUBA1B plays multifaceted oncogenic roles, influencing cytoskeletal remodeling, protein degradation, immune cell infiltration, tumor growth, chemoresistance, apoptosis, and patient survival across various malignancies.<br>• TUBA1B's expression levels were significantly elevated in several cancer types, including BC, compared to the corresponding healthy tissue levels [289].<br>• TUBA1B has been identified as a central gene involved in aggrephagy—a specialized type of lysosome-dependent autophagy that targets and removes misfolded or aggregated proteins [289]. These aberrant proteins, often arising from mutations or cellular stress, are marked as defective and must be cleared to avoid toxic buildup. Normally, the Ub–proteasome system handles the breakdown of such proteins; however, when protein aggregation occurs, the Ub-proteasome system may be unable to process them efficiently. In these situations, aggrephagy serves as an alternative degradation mechanism, serves to clear misfolded protein condensates, and contributes to cancer cell homeostasis [289].<br>• High TUBA1B expression in glioma is linked to increased cell proliferation, migration, autophagy, and apoptosis. Immune profiling indicates an association with cancer-associated fibroblasts and diverse immune cell infiltrates, suggesting a role in shaping the tumor microenvironment. TUBA1B-high tumors exhibit significant upregulation of cell cycle–related pathways and genes. These findings indicate that TUBA1B influences glioma biology through (i) intrinsic cell cycle regulation, (ii) enabling cancer cells to better cope with proteotoxic stress, and (iii) by modulating the immune milieu [289].<br>• TUBA1B overexpression likely fuels tumor aggressiveness by simultaneously driving intrinsic tumor growth programs, sustaining therapy-resistant populations, and shaping an immune-suppressive, tumor-promoting microenvironment—while bolstering the cell's ability to cope with stress through enhanced proteostasis mechanisms.

Suppl. Table S4: Frequency of gene amplification of the indicated genes, detected among breast invasive carcinoma tumors.
The datasets combined for the above-mentioned analysis (https://www.cbioportal.org/datasets), samples sizes, and their relevant citations are provided in the panel below.

**Gene**	**Percentage of breast invasive carcinoma tumors showing copy number alterations (gene amplification)**

FOXM1	2.5
WDR5	0.6
ASPM	13.3
KIF11	0.01
KIF14	13.5
KIF20A	0.4
KIF2C	1.0
KIF4A	0.1
CENPA	0.4
CENPF	14.4
CENPO	0.5
CENPL	11.9
OIP5	0.3
UBE2S	2.4
UBE2C	3.2
UBE2T	14.4
PSMD14	0.1
TUBA1B	0.3

cBioportal Dataset analyzed	Sample Size	Citation
METABRIC	2509	Curtis, C.; Shah, S.P.; Chin, S.F.; Turashvili, G.; Rueda, O.M.; Dunning, M.J.; Speed, D.; Lynch, A.G.; Samarajiwa, S.; Yuan, Y.; et al. The genomic and transcriptomic architecture of 2,000 breast tumours reveals novel subgroups. *Nature* **2012**, *486*, 346–352. https://doi.org/10.1038/nature10983<br><br>Pereira, B.; Chin, S.F.; Rueda, O.M.; Vollan, H.K.M.; Provenzano, E.; Bardwell, H.A.; Pugh, M.; Jones, L.; Russell, R.; Sammut, S.J.; et al. The somatic mutation profiles of 2,433 breast cancers refine their genomic and transcriptomic landscapes. *Nat. Commun.* **2016**, *7*, 11479. https://doi.org/10.1038/ncomms11479.
MSK	3879	Zehir, A.; Benayed, R.; Shah, R.H.; Syed, A.; Middha, S.; Kim, H.R.; Srinivasan, P.; Gao, J.; Chakravarty, D.; Devlin, S.M.; et al. Mutational landscape of metastatic cancer revealed from prospective clinical sequencing of 10,000 patients. *Nat. Med.* **2017**, *23*, 703–713. https://doi.org/10.1038/nm.4333<br>(Still an evolving paper)
TCGA, Cell 2015	1635	Hoadley, K.A.; Yau, C.; Wolf, D.M.; Cherniack, A.D.; Tamborero, D.; Ng, S.; Leiserson, M.D.M.; Niu, B.; McLellan, M.D.; Uzunangelov, V.; et al. Multiplatform analysis of 12 cancer types reveals molecular

		classification within and across tissues of origin. *Cell* **2014**, *158*, 929–944. https://doi.org/10.1016/j.cell.2014.06.049
TCGA, Firehose Legacy	1896	Broad Institute TCGA Genome Data Analysis Center. Analysis Overview for TCGA Firehose Legacy Data Freeze 2016_01_28.
TCGA, Nature 2012	1136	The Cancer Genome Atlas Network. Comprehensive molecular portraits of human breast tumours. *Nature* **2012**, *490*, 61–70. https://doi.org/10.1038/nature11412
TCGA, PanCancer Atlas	1084	Hoadley, K.A.; Yau, C.; Hinoue, T.; Wolf, D.M.; Lazar, A.J.; Drill, E.; Shen, R.; Taylor, A.M.; Cherniack, A.D.; Thorsson, V.; et al. Cell-of-origin patterns dominate the molecular classification of 10,000 tumors from 33 types. *Cell* **2018**, *173*, 291–304.e6. https://doi.org/10.1016/j.cell.2018.03.022
TCGA GDC, 2025	1102	304. Grossman, R.L.; Heath, A.P.; Ferretti, V.; Varmus, H.E.; Lowy, D.R.; Kibbe, W.A.; Staudt, L.M. Toward a shared vision for cancer genomic data. *N. Engl. J. Med.* **2016**, *375*, 1109–1112. https://doi.org/10.1056/NEJMp1607591
