# Supplementary figures and images for: FOXM1 Signaling Network Transcriptionally Upregulates Expression of Proteins Involved in Mitotic Progression to Induce High Proliferation and Chromosomal Instability in Androgen Receptor-Low Triple-Negative Breast Cancer
Source: Int J Mol Sci. 2026 Feb 14;27(4):1823. doi: 10.3390/ijms27041823 (PMC12940972; doi:10.3390/ijms27041823)

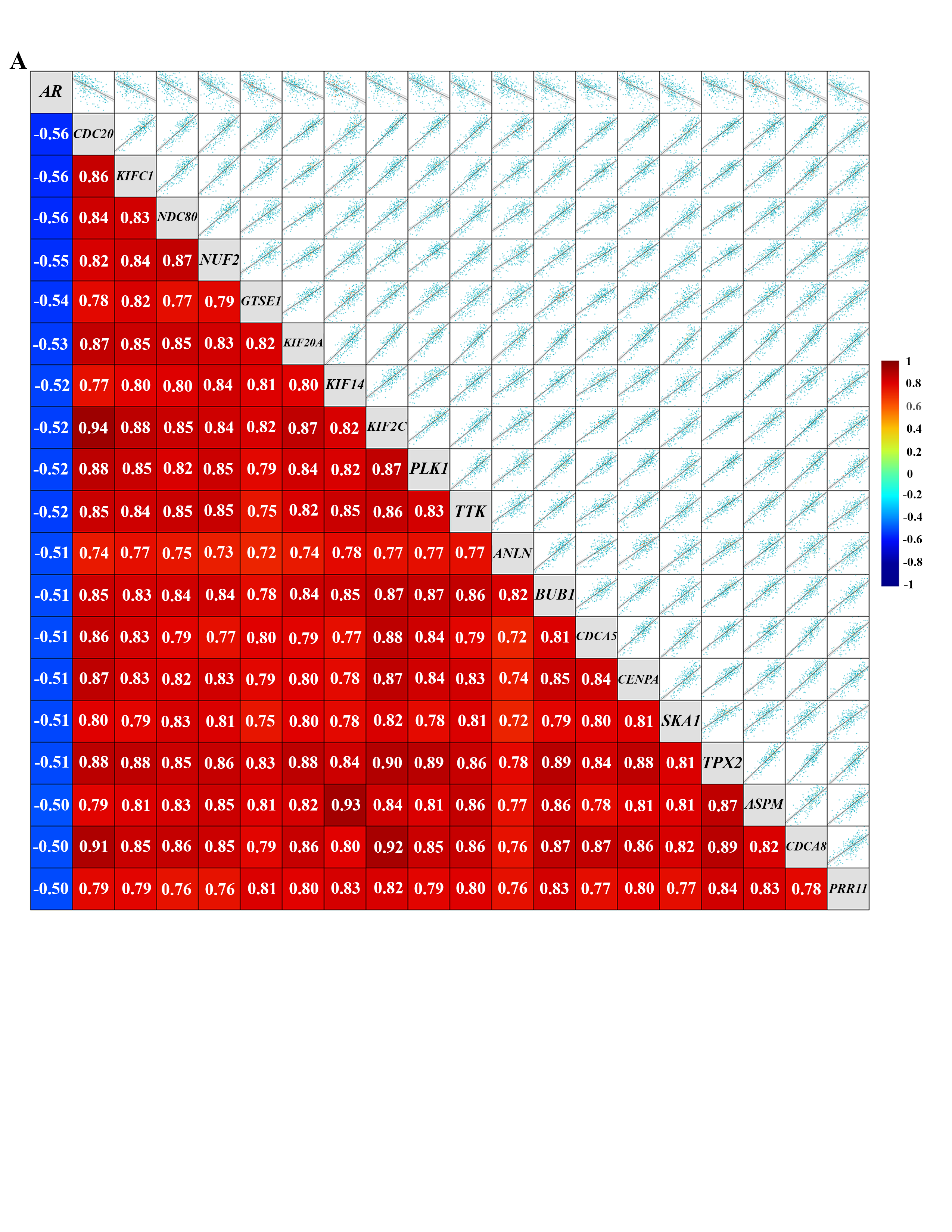

Supplement: Supplementary file 1 [file ijms-27-01823-s001.zip › Suppl Figure S1A—FLAT.tif]

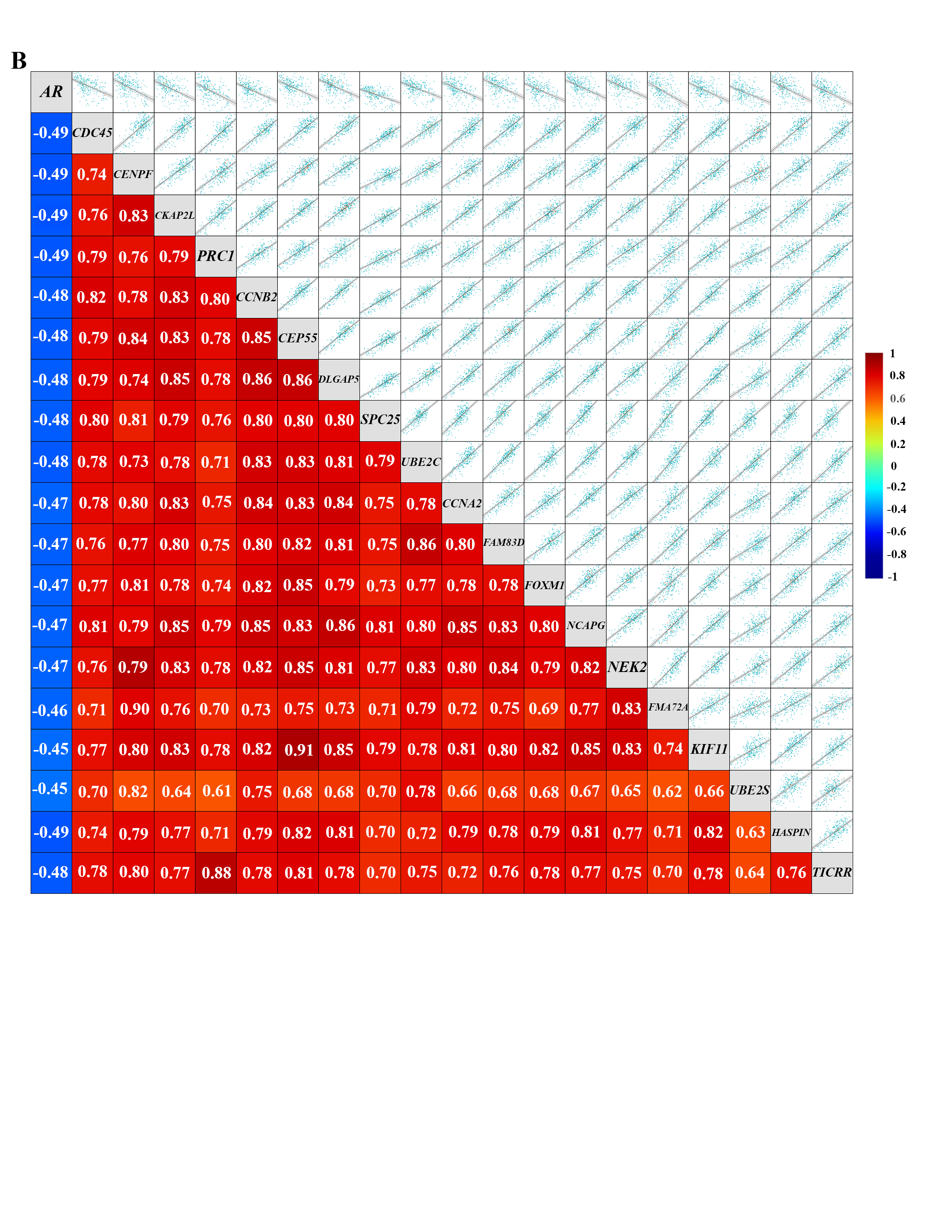

Supplement: Supplementary file 1 [file ijms-27-01823-s001.zip › Suppl Figure S1B—FLAT.tif]

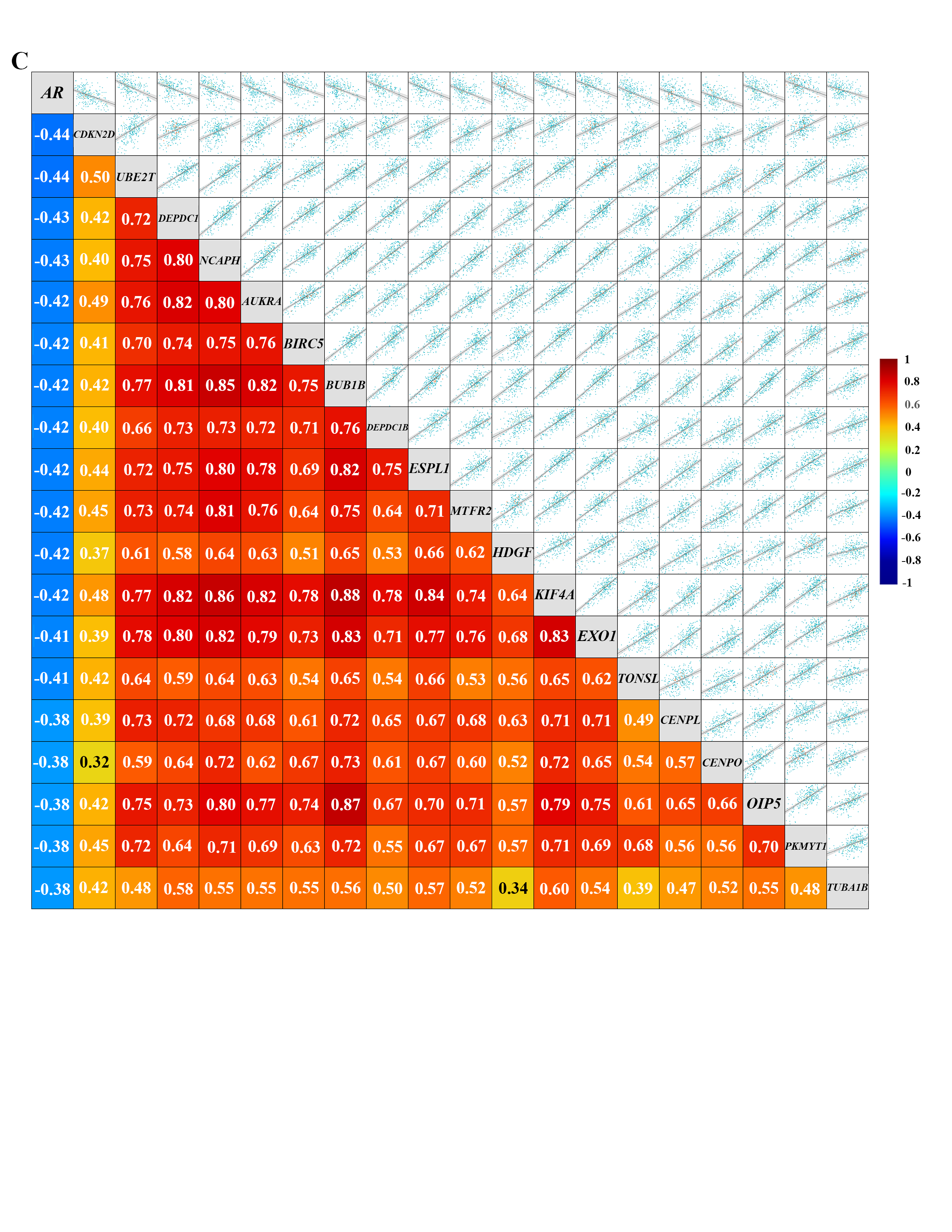

Supplement: Supplementary file 1 [file ijms-27-01823-s001.zip › Suppl Figure S1C—FLAT.tif]

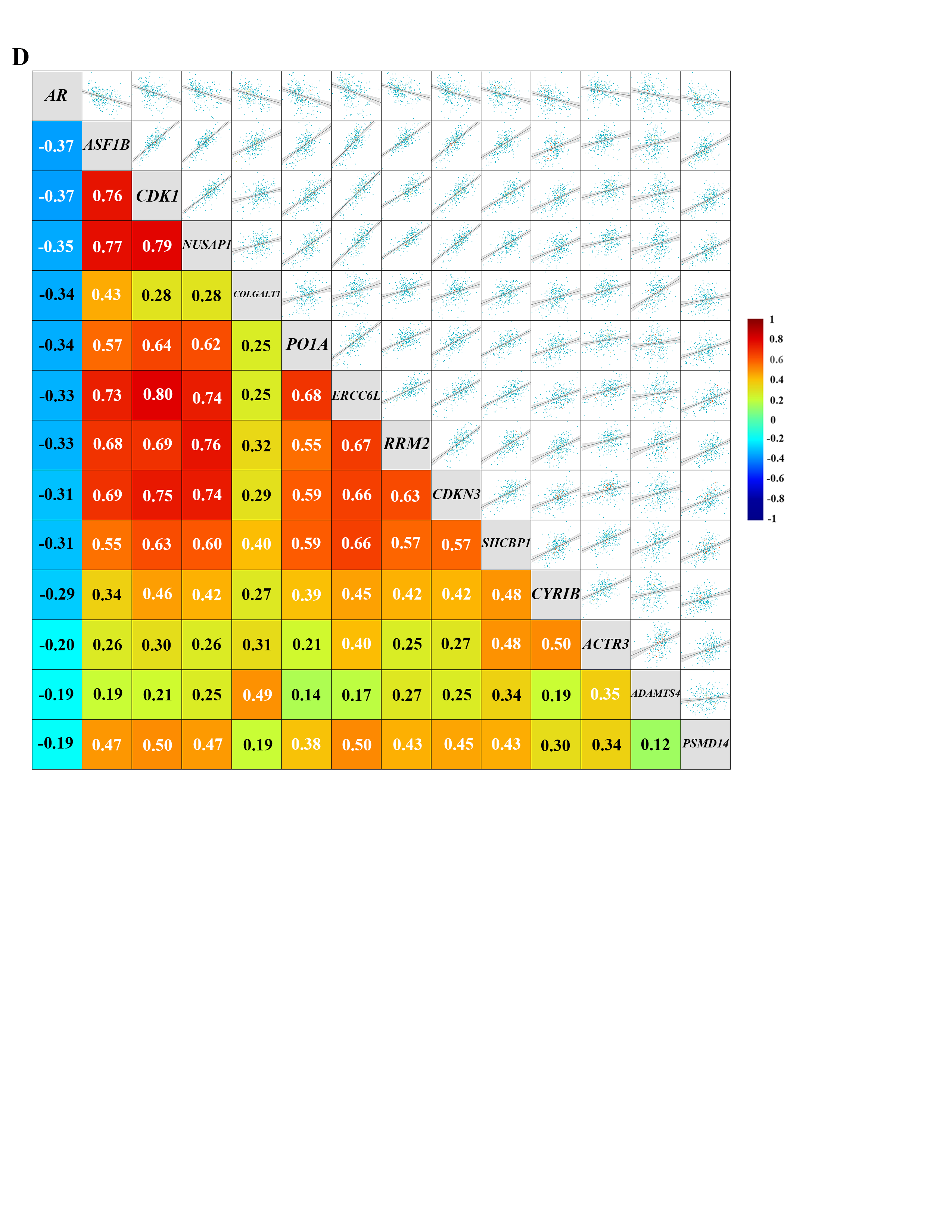

Supplement: Supplementary file 1 [file ijms-27-01823-s001.zip › Suppl Figure S1D—FLAT.tif]

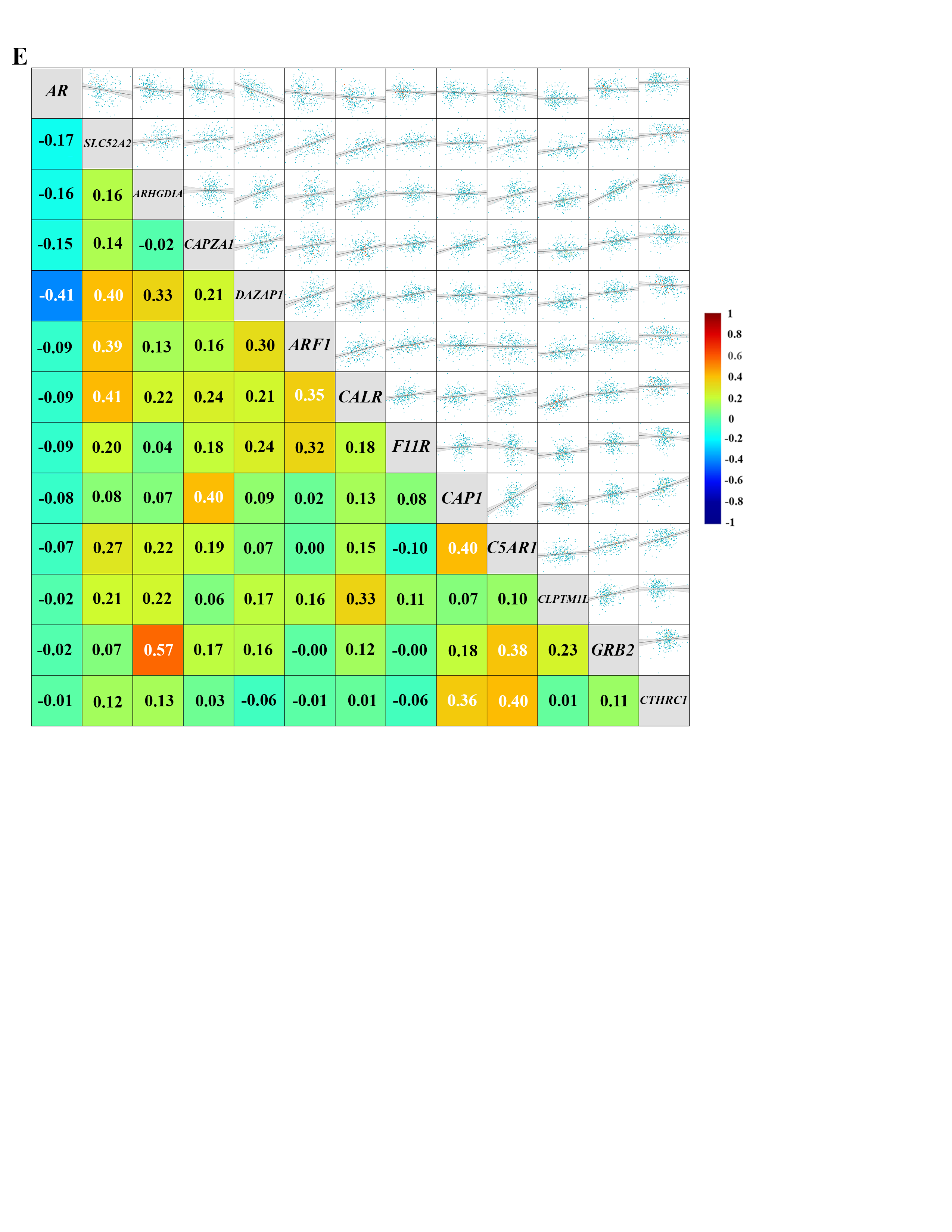

Supplement: Supplementary file 1 [file ijms-27-01823-s001.zip › Suppl Figure S1E—FLAT.tif]

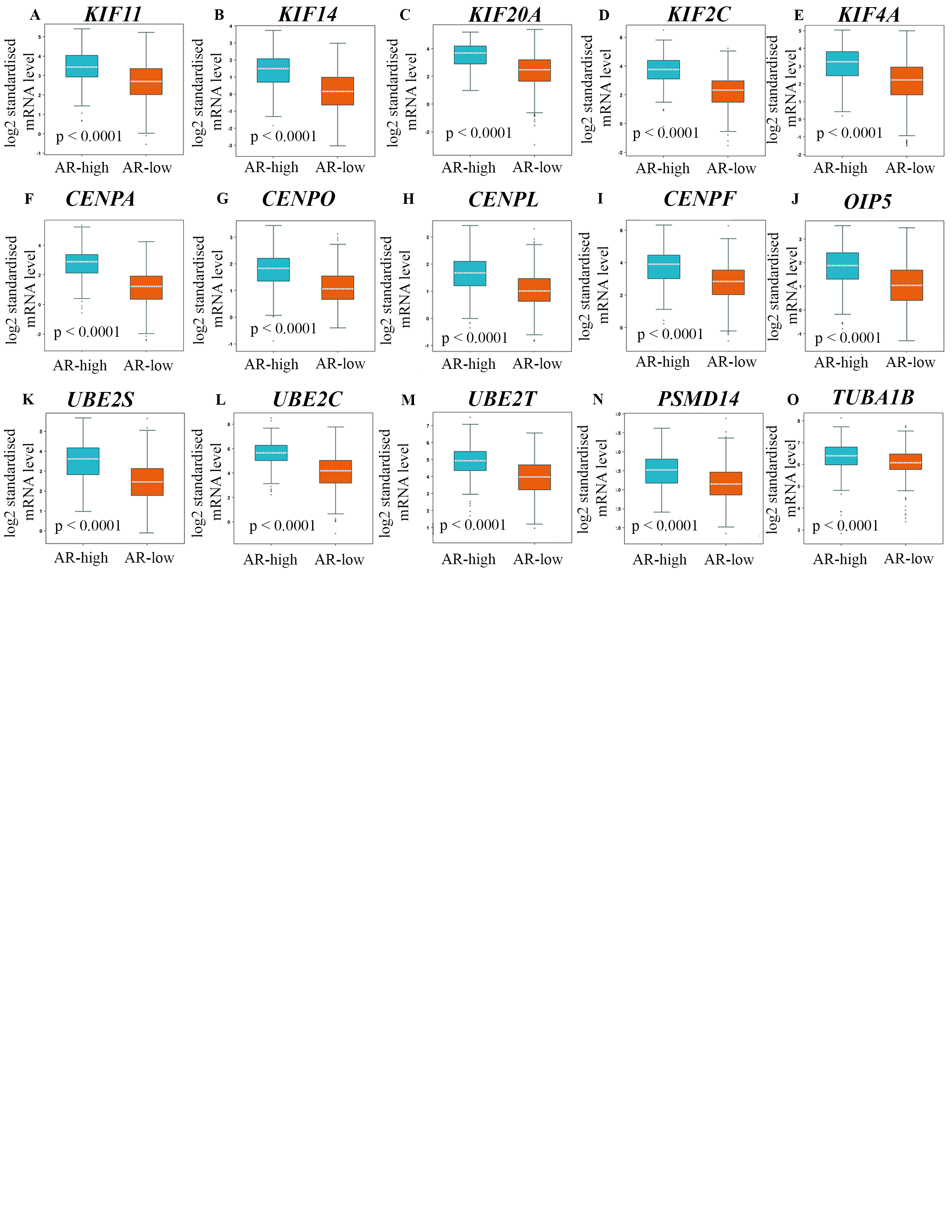

Supplement: Supplementary file 1 [file ijms-27-01823-s001.zip › Suppl Figure S2-AR—FLAT.tif]

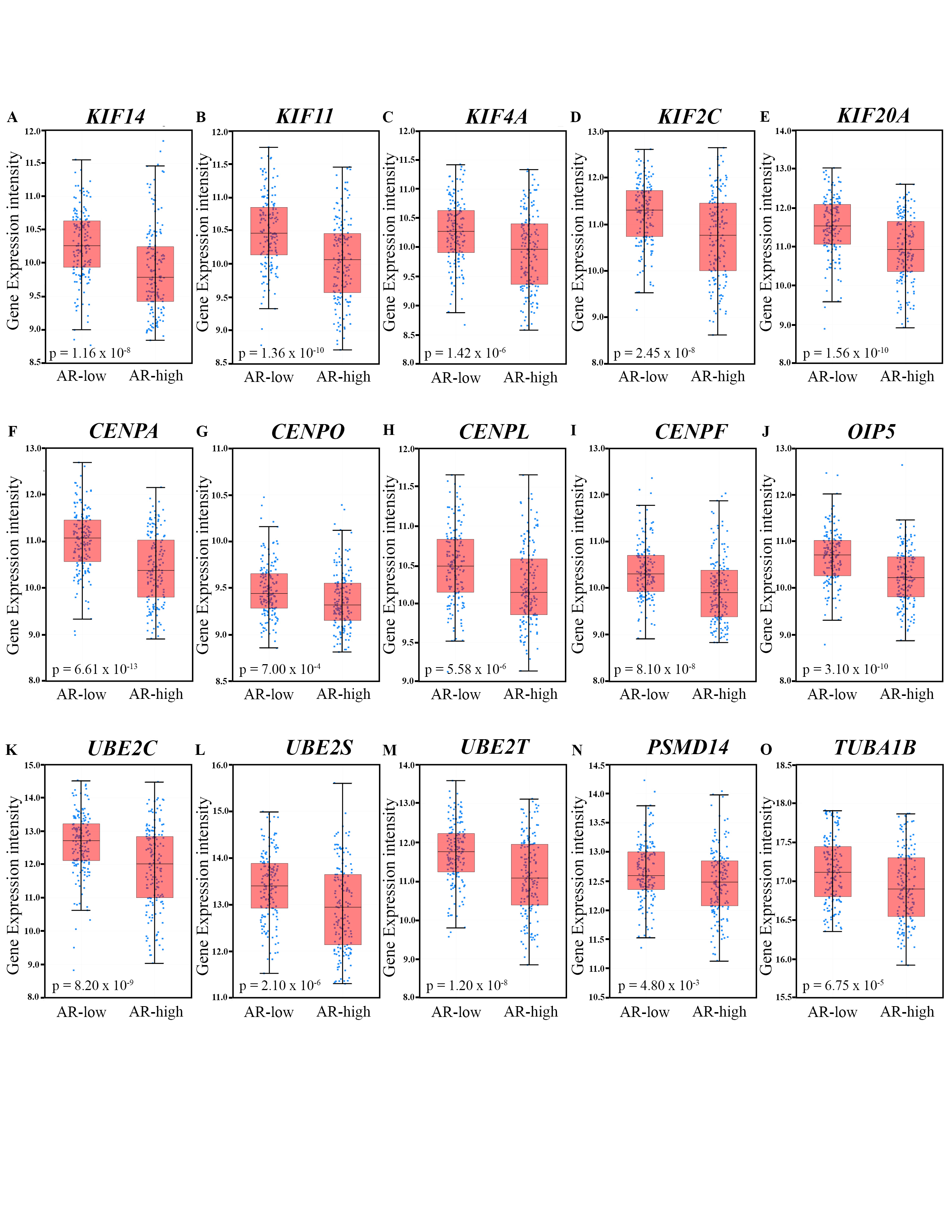

Supplement: Supplementary file 1 [file ijms-27-01823-s001.zip › Suppl Figure S3-METABRIC-revised.jpg]

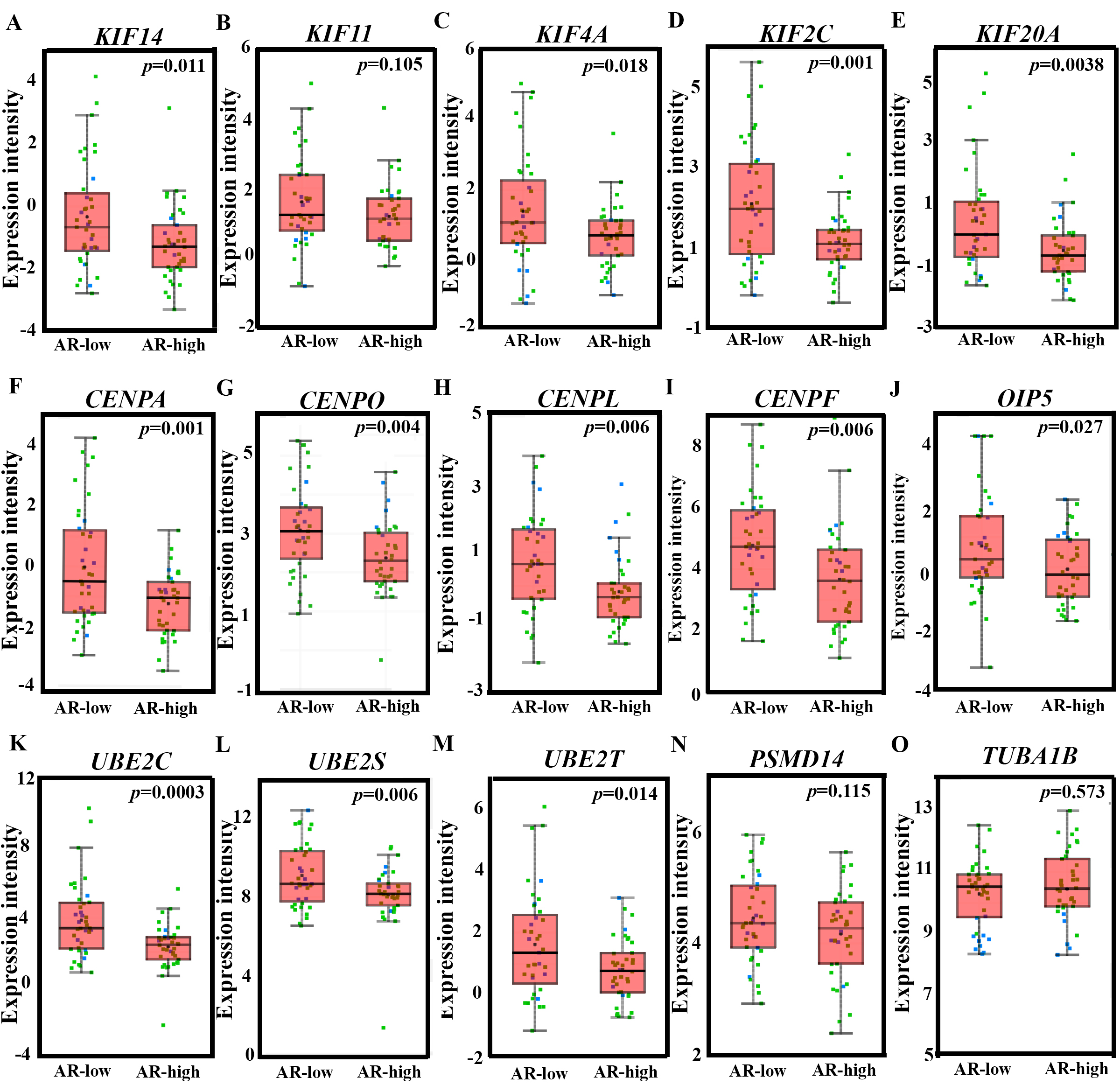

Supplement: Supplementary file 1 [file ijms-27-01823-s001.zip › Suppl Figure S4—ONCOHUMAN-revised.jpg]

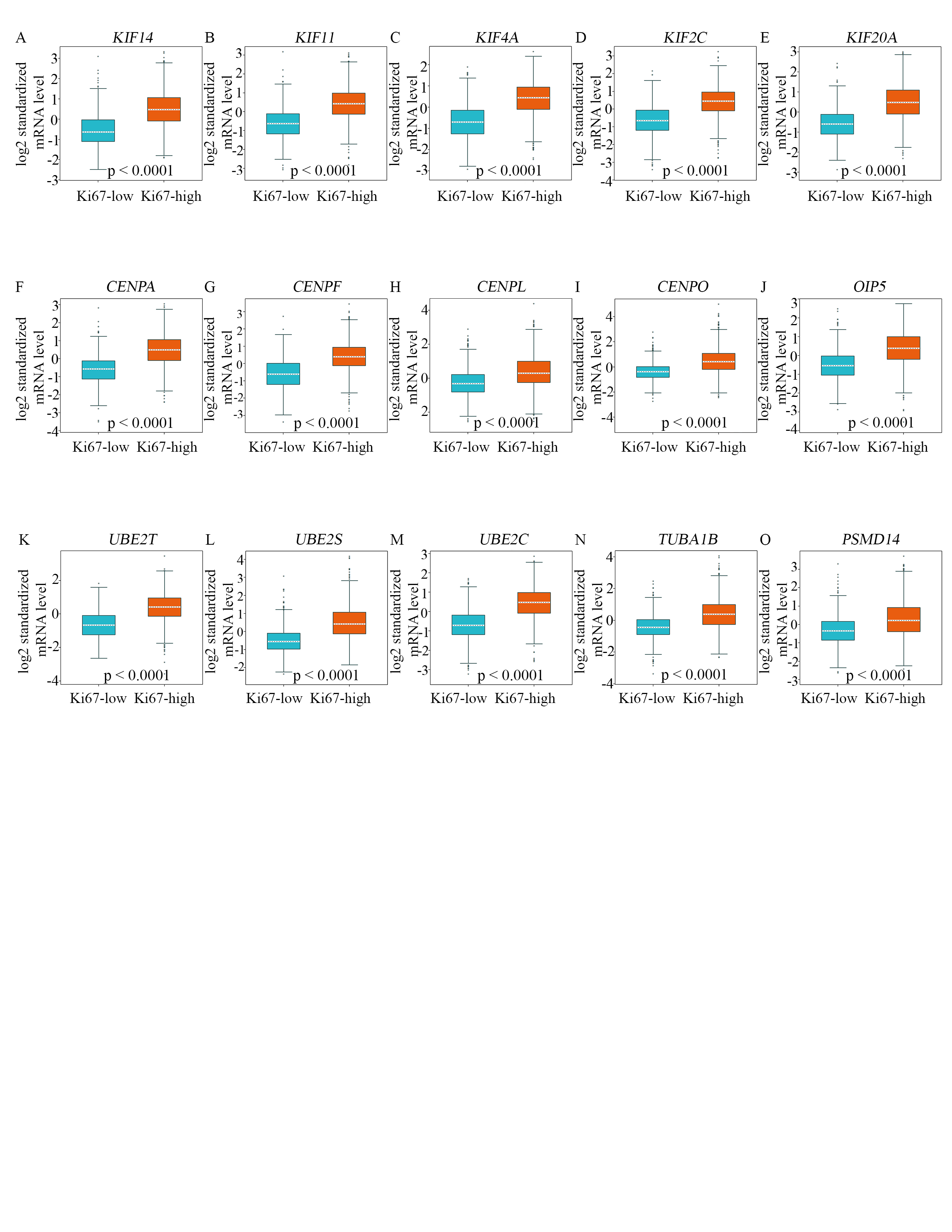

Supplement: Supplementary file 1 [file ijms-27-01823-s001.zip › Suppl Figure S5-Ki67—FLAT.tif]

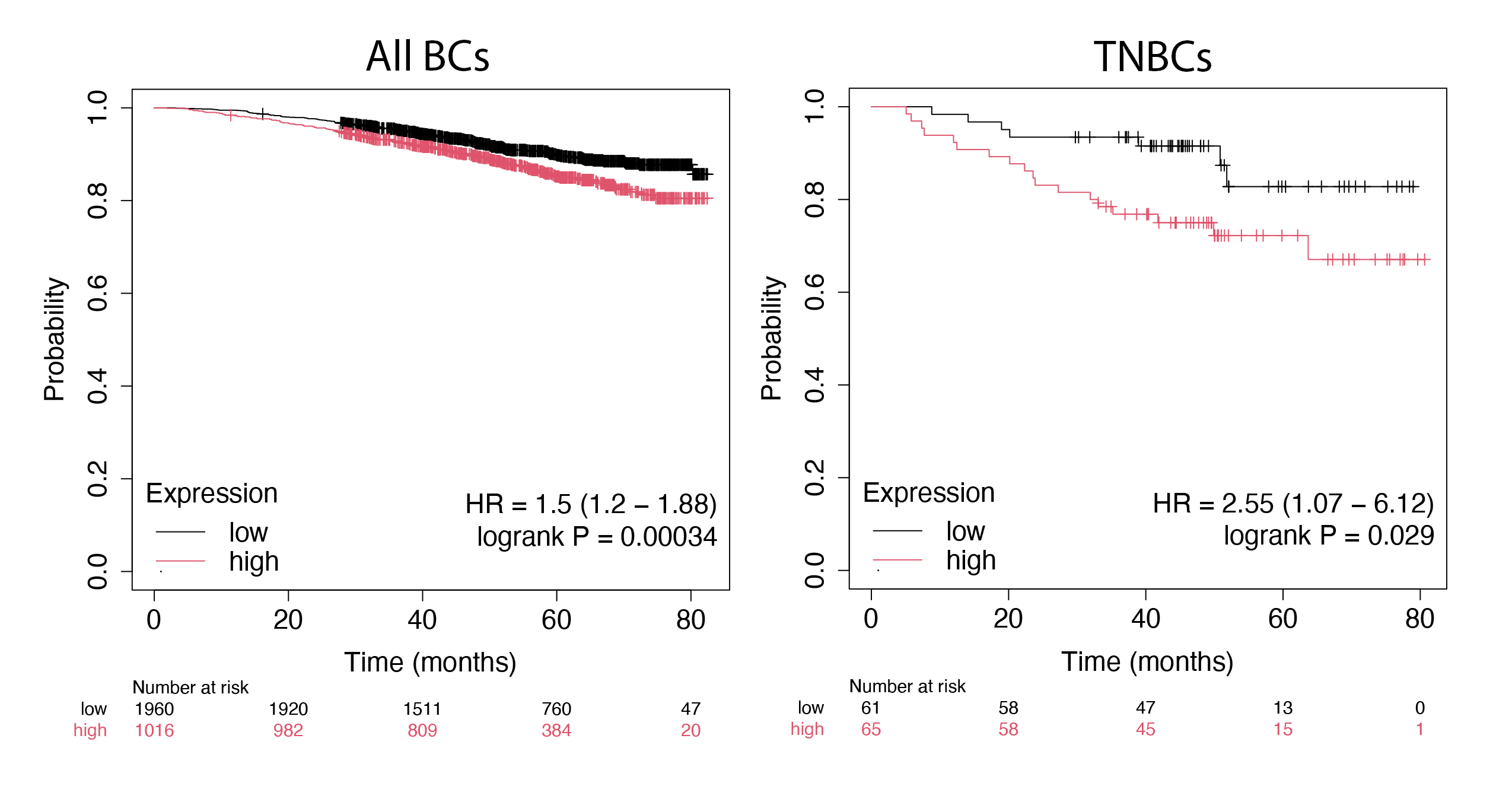

Supplement: Supplementary file 1 [file ijms-27-01823-s001.zip › Suppl Figure S6-Gene signature.jpg]
